# Root and canopy traits and adaptability genes explain drought tolerance responses in winter wheat

**DOI:** 10.1371/journal.pone.0242472

**Published:** 2021-04-05

**Authors:** A. S. Nehe, M. J. Foulkes, I. Ozturk, A. Rasheed, L. York, S. C. Kefauver, F. Ozdemir, A. Morgounov

**Affiliations:** 1 International Maize and Wheat Improvement Center (CIMMYT), Ankara, Turkey; 2 Division of Plant and Crop Science, School of Biosciences, University of Nottingham, Nottingham, United Kingdom; 3 International Maize and Wheat Improvement Center (CIMMYT) China Office, Beijing, China; 4 Noble Research Institute, Ardmore, Oklahoma, United States of America; 5 Integrative Crop Ecophysiology Group, University of Barcelona, Barcelona, Spain; 6 Bahri Dagdas International Agricultural Research Institute, Konya, Turkey; 7 Omsk State Agrarian University, Omsk, Russia; CSIR-Institute of Himalayan Bioresource Technology, INDIA

## Abstract

Bread wheat (*Triticum aestivum* L) is one of the three main staple crops worldwide contributing 20% calories in the human diet. Drought stress is the main factor limiting yields and threatening food security, with climate change resulting in more frequent and intense drought. Developing drought-tolerant wheat cultivars is a promising way forward. The use of holistic approaches that include high-throughput phenotyping and genetic markers in selection could help in accelerating genetic gains. Fifty advanced breeding lines were selected from the CIMMYT Turkey winter wheat breeding program and studied under irrigated and semiarid conditions in two years. High-throughput phenotyping was done for wheat crown root traits and canopy senescence dynamics using vegetation indices (green area using RGB images and Normalized Difference Vegetation Index using spectral reflectance). In addition, genotyping by KASP markers for adaptability genes was done. Overall, under semiarid conditions yield reduced by 3.09 t ha^-1^ (-46.8%) compared to irrigated conditions. Genotypes responded differently under drought stress and genotypes 39 (VORONA/HD24-12//GUN/7/VEE#8//…/8/ALTAY), 18 (BiII98) and 29 (NIKIFOR//KROSHKA) were the most drought tolerant. Root traits including shallow nodal root angle under irrigated conditions and root number per shoot under semiarid conditions were correlated with increased grain yield. RGB based vegetation index measuring canopy green area at anthesis was better correlated with GY than NDVI was with GY under drought. The markers for five established functional genes (*PRR73*.*A1* –flowering time, *TEF-7A* –grain size and weight, *TaCwi*.*4A* - yield under drought, *Dreb1*- drought tolerance, and *ISBW11*.*GY*.*QTL*.*CANDIDATE*- grain yield) were associated with different drought-tolerance traits in this experiment. We conclude that–genotypes 39, 18 and 29 could be used for drought tolerance breeding. The trait combinations of canopy green area at anthesis, and root number per shoot along with key drought adaptability makers (*TaCwi*.*4A* and *Dreb1*) could be used in screening drought tolerance wheat breeding lines.

## 1. Introduction

Wheat (*Triticum aestivum* L.) is one of the most important food crops contributing around 20% of calories in the human diet worldwide. However, climate change has resulted in more frequent and intense periods of drought which affect wheat production [1]. Worldwide drought is the most important factor affecting wheat yields [2] and model-based predictions indicate that there will be 9–12% wheat yield reduction with climate change in 21^st^ century without considering the benefits of CO_2_ fertilization and adaptations [3]. Developing new drought-tolerant varieties is therefore important to achieve food security in the context of climate change. Identifying drought-tolerance traits for deployment in breeding is therefore crucial. A deeper root system and cooler canopy temperature are two important traits reported to be correlated with for drought tolerance in wheat [4–6]. As roots are difficult to study under field conditions, canopy temperature has been applied as an indirect way of assessing the root depth as higher water uptake and leaf transpiration is related to a cooler canopy. Canopy stay-green characters have also shown promise to help select drought-tolerant genotypes as they may confer extended photosynthesis, nutrient and water uptake under stress conditions [7, 8].

Wheat root systems consist of seminal roots (up to 6) and crown roots (around 10–15) per plant the latter emerging from basal node of main shoots and tillers [9]. These two root systems function together to acquire water and nutrients from the soil [10–12]. Distribution of root length density (root length per unit soil volume; RLD) with depth is an important trait affecting water capture in wheat crops [7, 10, 13]. In synthetic wheat-derived lines, yield increase under water stress conditions was correlated with increase in root dry weight at depth in NW Mexico [14]. Higher allocation of plant assimilates to deeper roots has been correlated with a cooler canopy and increase in overall grain yield under drought conditions in wheat synthetic-derived material [5]. Narrower root angle in the top soil (steeper roots) has correlated with higher root density in deeper soil in wheat genotypes in Australia [14–16].

The high-throughput phenotyping of root system architecture under the field conditions presents a bottleneck in breeding for drought tolerance in wheat [17]. Previously, the soil-core break method [18] and ‘shovelomics’ [19] have been used for high-throughput field phenotyping in cereals. The core-break method involves the counting of roots visible on the cross-section to estimate root length density [20]. Shovelomics, focuses on crown root phenotyping, and involves the excavation of roots in the topsoil and measuring root traits manually or through image analysis. Results of direct measurements and visual scoring in maize showed correlations with root depth for crown root number and angle [19]. Shovelomics methods have quantified genetic variation in crown root angle and root length in maize [19, 21, 22], barley [23] and durum wheat [24]. We used a high-throughput shovelomics technique for phenotyping root crown architecture of the whole root crown in wheat.

The use of Normalized Difference Vegetation Index (NDVI) spectral reflectance index to study canopy growth and senescence dynamics is well established, but has some limitations [25, 26]. Especially at high values of leaf area index (LAI), NDVI tends to saturate and does not show strong linear association with yield components [27, 28]. Also, in order to obtain accurate results with NDVI, bright conditions with direct sunlight are required while taking the measurements (in the case of passive sensors). Senescence is a genetically programmed and environmentally influenced process [29] and the stay-green phenotype has been shown to be improving yields under drought [8, 30]. NDVI has been used to measure stay green trait in wheat under drought [8]. More recently, RGB image-based vegetation indices have proved to be better correlated with grain yield than NDVI under similar circumstances and also are time-saving [28].

Marker-assisted selection is a very important component of molecular breeding to develop resilient cultivars by selecting and accumulating favorable alleles. In bread wheat several genes underpinning drought tolerance have been identified and molecular markers have been developed to select favorable alleles [31, 32]. However, the distribution and association of alleles for functional genes like *Dreb1*, *PRR-73*, *TaCwi-A1* and *TEF-7* associated with drought tolerance is largely unknown in wheat cultivars from the most parts of the world. Transcription factors like *DREB* control the expression of several functional genes responsible for plant tolerance to drought, and have been proposed for use in plant improvement for drought tolerance [33]. Similarly, *TaTEF-7A* is transcript elongation factor gene responsible for grain number per spike [34], thousand grain weight and chlorophyll content at grain-filling stage under drought stress [35]. The gene *TaPRR73* was found to be regulation of flowering date and can be used in breeding to develop cultivars adaptable for different geographical areas [36]. *TaCwi-A1* gene produce cell wall invertase enzyme mainly responsible for sink tissue development and carbon allocation and also showed affecting grain weight and potential role in drought tolerance in wheat [37, 38].

The present study reports associations between nodal root traits measured using the wheat shovelomics techniques along with vegetation indices in a set of 50 CIMMYT Turkey winter wheat cultivars and advanced lines. The experiment was conducted under irrigated (IR) and semiarid (SA) field conditions in Turkey in two cropping seasons. The germplasm was also screened for allelic variation of genes previously related to drought adaptability genes using breeder friendly KASP markers. Our aim was to quantify genetic variation in grain yield responses to drought and its physiological basis and to identify drought adaptability genes for potential use in marker-assisted selection for drought tolerance in wheat.

## 2. Materials and methods

### 2.1 Experimental design and plot management

Two field experiments were sown on 15 Nov 2017 and 8 Nov 2018 at Bahri Dagdas International Agricultural Research Institute, Konya in 2017–18 and 2018–19 wheat growing season. Before sowing the experimental field was fallow. The soil type was sandy clay. Experiments were conducted in a randomized block, split–plot design, in which two irrigation treatments (IR: drip-irrigated and SA: semiarid/rain-fed) were randomized on main-plots, and 50 CIMMYT winter wheat cultivars and advanced lines including 4 check cultivars were randomized on sub-plots in two replicates. The 50 winter wheat genotypes represent modern germplasm developed by the CIMMYT-International Winter Wheat Improvement Program and obtained from cooperators in Eastern Europe. These lines were selected from 100 genotypes tested in previous years and represented three groups: high yielding under irrigation; high yielding under drought and balanced performance under both environments. The check cultivars used were Gerek (widely grown drought resistant check), Katea (widely grown irrigated check), Konya (high yield potential irrigated check) and Nacibey (check for supplementary irrigation) (S1 Table). Plots were 7.0 m × 1.2 m with 6 rows 20 cm apart and 450 seeds were sown per square meter. Fertilizers applied were 100 kg ha^−1^ of phosphorus (P) and 140 kg ha^−1^ of nitrogen (as ammonium nitrate) per hectare at the time of planting, and an additional 50 kg ha^−1^ of N at tillering (GS35). Under the irrigated treatment drip-irrigation was given as 50 mm application each time. Irrigation was given twice during the crop growth season at the tillering and flowering stage. The irrigation was applied according to past experience at the field site so that under irrigated conditions there would not be significant water stress.

### 2.2 Crop measurements: Grain yield and yield components

In 2018, a 1.5 m row bulk sample was hand-harvested by cutting at ground level at physiological maturity (GS89) [39]. The fertile shoots (those with an ear) were counted and 5 primary (large ear and stem) fertile shoots were selected for dry matter partitioning analysis. In 2019, 10–20 shoots were selected for dry matter partitioning analysis from the sample taken for root traits analysis (explained in next section). All selected shoots were separated into ears and straw. Dry weight of the ears and the straw was recorded after drying at 80°C for 48 h. The ears of the bulk sample were then hand threshed and grain weighed. All grains were counted by a Contador seed counter (Pfeuffer, Germany) and thousand-grain weight (TGW) was calculated. From these data the grain DM per fertile shoot, harvest index (HI; grain DM / above-ground DM), fruiting efficiency (grain weight per ear dry weight) and above ground dry matter (AGDM; GY/HI) were calculated. The grain yield was calculated by weighing grain from rest of the plot which was machine-harvested (adjusted to 85% dry weight).

### 2.3 Shovelomics root crown trait measurements

The methodology for root excavation was the same in both the years whereas root traits were assessed in different ways for each years. Root crowns were excavated from all sub-plots during late-grain filling. A spade of 25 cm width and 30 cm depth was inserted to 20 cm depth on either side of plants keeping the blade parallel to the row. A single sample was taken per plot. The soil was placed into a 10 L bucket filled with water overnight. The next day the root crowns were sprayed with low pressure water from a hose to remove remaining soil. Three plants per sample were selected for scanning or image analysis. In 2018, root images were acquired and analyzed using WinRHIZO Regular V. 2009c scanner and software (Regent Instruments Inc., Canada). The traits measured were root surface area (cm^2^), root diameter (mm), and root volume (cm^3^) per plant. In 2019, images of the roots were taken with a RGB camera (Sony a 6000). A single image per sample was taken with auto setting. Roots were placed on a black background to maximum contrast and sample ID label and reference scale (white square of 2 cm x 1 cm) was placed on one side of the roots as shown in S1 Fig. Images were analyzed using a modified method from York and Lynch [40]. A project for the ObjectJ plugin (https://sils.fnwi.uva.nl/bcb/objectj) for ImageJ [41] was created to allow the angles, numbers, stem diameter and roots diameter to be annotated and measured from the image of the plant-root samples (S1 Fig). The pixel dimensions were converted to physical units using measurements of the known-sized scale in every image. The traits measured were root number per shoot and plant, root diameter (mm), and root angle (°). A polyline as showed in S1 Fig was used to measure the crown lengths of the outermost roots. The seminal root length, and the angle was measured for the outermost crown roots at approximately 5 cm depth by measuring the width then later calculating angle using trigonometry and the actual depth measurement to where width was measured (S1 Fig). For nodal root number, each nodal root axis was manually annotated, and the count recorded in an output file. The image analysis gave values for the number of pixels corresponding to root diameter, length and numbers. Using the 2 cm x 1 cm reference square, these pixel values were then converted to the relevant units for each root measurement in Excel and the angles were calculated as detailed in York and Lynch [40]. The trait definitions for the shovelomics root system architecture traits measured in 2019 are given in S3 Table.

### 2.4 NDVI and RGB based vegetation indexes and canopy temperature

In 2018, Normalized Difference Vegetation Index (NDVI) was measured using the handheld active sensor Trimble GreenSeeker spectroradiometer (Trimble Navigation Ltd, USA) to assess the canopy green area starting from booting (GS41). Modified Gompertz curves (Eq 1) were fitted to the NDVI values against thermal time (base temp. 0°C after anthesis, GS61, [39]). The Gompertz (T) parameter was fitted as the thermal time for the NDVI to decrease to 37% of the NDVI value at GS61. The thermal time (t, measured in °CD) when NDVI values were 90% and 10% of the value at GS61 was taken as the onset of senescence (SenSt) and end of senescence (SenEnd), respectively, and the duration from 90% NDVI to 10% NDVI remaining was considered as the senescence duration (SenDu).

Y=K*exp{−exp((t−T)*2/D)} (1)

where t is thermal time (base temp. 0°C), D is the duration of senescence (SenDu), T is the timing of the inflection point at 37% NDVI value remaining from initial point at GS61, and K is the maximum NDVI at GS61. The senescence parameters were estimated for each sub-plot and then subjected to ANOVA.

In 2019, RGB images and NDVI (GreenSeeker Trimble Navigation Ltd, USA) were taken every two weeks from tillering (GS35) to crop maturity (GS89). RGB image-based vegetation index—green area per meter square (GA m^-2^) was calculated using Eqs 2 to 6:

GSD=(SWxH)/(FLxIW) (2)

DW=GSDxIW (3)

DH=GSDxIH (4)

A=DWxDH (5)

$${\text{Green}\;\text{area}\;\text{m}}^{-2}=\text{GA}\;\text{x}\;\text{A}$$ (6)

whereas GSD is ground sampling distance (centimeters/pixel), SW is camera sensor width (mm), H is camera height from top of the canopy (m), FL is focal length of camera (mm), DW is width of single image footprint on the ground (m), DH is height of single image footprint on the ground (m), IW is image width (pixels), IH is image height (pixels), A is ground area in image (m^2^) and GA is index value output after image analysis using BreedPix. BreedPix is open source software [42], implemented as part of the open-source CerealScanner plugin developed for ImageJ software [28, 41]. Green Area per meter square at anthesis (GA An) and 2 weeks after anthesis (GA 2W) values are used in this paper. Canopy temperature was measured at anthesis using handheld infrared temperature meter (SBRMART GM320).

### 2.5 Genotyping

DNA was extracted from all genotypes using a modified CTAB method [43]. Allele-specific KASP markers for five different loci were used. The primer sequences and amplification conditions of each gene are described in [38]. The detailed genotyping procedures have been described in previous studies [38, 44]. Briefly, two allele-specific primers carrying a standard FAM tail (5′-GAAGGTGACCAAGTTCATGCT-3′) and HEX tail (5′-GAAGGTCGGAGTCAACGGATT-3′), with targeting SNP at the 3′end, and a common reverse primer were synthesized. The primer mixture included 46 μl ddH_2_O, 30 μl common primer (100 μM) and 12 μl of each tailed primer (100 μM). Assays were tested in 384-well format and set up as 5 μl reaction [2.2 μl DNA (10–20 ng/μl), 2.5 μl of 2XKASP master mixture and 0.056 μl primer mixture]. PCR cycling was performed using the following protocol: hot start at 95°C for 15 min, followed by ten touchdown cycles (95°C for 20 s; touchdown 65°C and decreasing by –1°C per cycle for 25 s) further followed by 30 cycles of amplification (95°C for 10 s; 57°C for 60 s). The extension step was not required because amplicon size is less than 120 bp. The plate was read in the BioTek H1 system and data analysis was performed manually using Klustercaller software (version 2.22.0.5; LGC Hoddesdon, United Kingdom).

### 2.6 Marker-traits association analysis

In this paper we presented results for five key KASP markers out of 150 for their association with phenotypes (Table 4). KASP markers for which one of the alleles was represented at relatively higher frequency (>80%) than other alleles were not considered for MTA. These markers are regularly used in marker-assisted selection in CIMMYT’s wheat breeding program. As some of the traits that we measured differed between years, the marker-traits associations (MTAs) were identified separately for each year. MTA analysis was done in R using a linear model (lm) function (Eq 7) to test the significant effect of the marker on traits by comparing the mean. BLUEs (best linear unbiased estimators) were calculated using Meta-R for a randomized block design for all the traits for individual years. BLUE values were used to test the significant effect of the marker on traits using following liner model.

$${\text{Y}}_{\text{jk}}=\mu +{\text{M}}_{\text{j}}+{\text{G}}_{\text{k}}({\text{M}}_{\text{j}})$$ (7)

Y is phenotyping value, μ is mean of the population, M is mean effect of j^th^ marker, G_k_(M_j_) genotype within marker variance (error variance).

### 2.7 Statistics

In both years, GenStat 19th edition (VSN International, Hemel Hempstead, UK) was used for carrying out analysis of variance (ANOVA) of traits applying a split-plot design with replications and genotypes regarded as random effects and the least significant difference (LSD) test was used to compare the means between specific treatments. A cross-year ANOVA was applied to analyze irrigation treatments and genotypes effects across years and the interaction with year, assuming irrigation treatments and genotypes were fixed effects and replicates and year were random effects. Pearson’s correlation coefficient (r) and the linear regressions coefficient (R^2^) were calculated to quantify associations between traits for mean values in individual years and cross year means using GenStat 19^th^ editions. Principal component analyses were done to produce biplots using R software package “factoextra.”

Forward stepwise multi-linear regression was applied to 50 genotypes for each treatment and year separately with GY as the dependent variables and root surface area (RoSuAr), root diameter (RoDiM), root volume (RoVol), NDVI at anthesis (NDVI), NDVI senescence start (SenSt), NDVI senescence duration (SenDu) as independent variables in 2018 and root angle (RoAng), root diameter (RoDiM), root dry weight per shoot (RoDrWtSh), canopy green area per meter square at anthesis (GA An) and NDVI at anthesis (NDVI) in 2019 using GenStat 19th Editions (VSN International 2017). The R^2^ statistic values are presented calculated as: 100 × (1 –(residual mean square/total mean square)).

## 3. Results

### 3.1 Drought effects on plant growth

**Fig 1:**
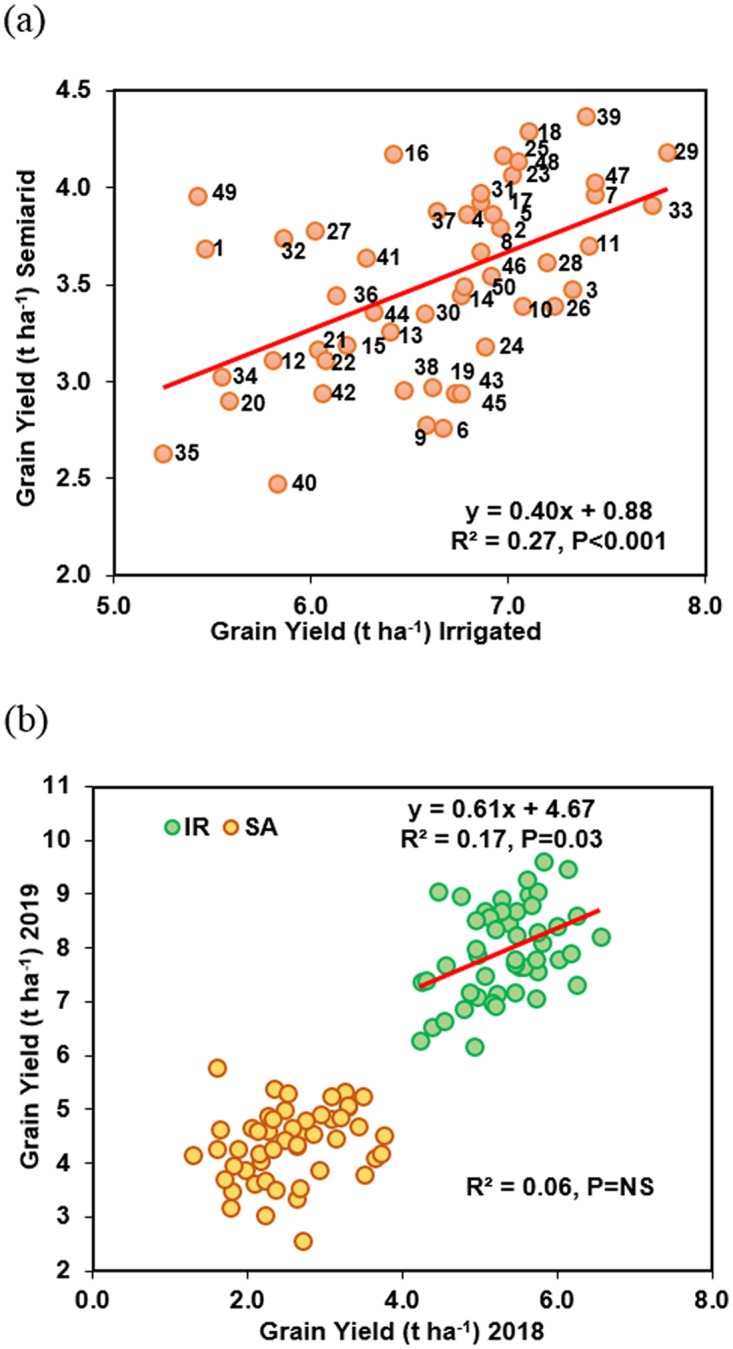
Linear regression among the 50 winter wheat genotypes for (a) grain yield in semiarid on GY in irrigated conditions (mean across the years) and (b) GY in 2018 on GY in 2019 under Irrigated (IR) and semiarid (SA) conditions.

Averaging across the 50 genotypes, the drought/semiarid (SA) conditions reduced the grain yield (GY) compared to irrigated (IR) conditions by 2.67 t ha^-1^ (-50.1%) in 2018 and 3.51 t ha^-1^ (-44.6%) in 2019 (*P* < 0.001; Table 1) with an average reduction over two years of 3.09 t ha^-1^ (-46.8%, *P* = 0.01, Fig 1). The cross-year ANOVA showed a significant Year x Genotype (Y x G) interaction (*P*<0.001, Table 1). Relative loss in GY under SA conditions ranged from -36.1% (genotype code no. 32) compared to -58.5% (genotype 9). The three-way interaction of Y x T x G (Year x Treatment x Genotype) was not significant.

**Table 1:** Yield components traits for 50 CIMMYT winter wheat genotypes for 2018, 2019 and cross-year means (CY).

	GY (t ha^-1^)		BM (t ha^-1^)		HI		PH (cm)		HD		TGW (g)		FE grains (g^-1^)	
Genotype	IR	SA	IR	SA	IR	SA	IR	SA	IR	SA	IR	SA	IR	SA
Mean 2018	5.33	2.67	12.9	5.97	0.42	0.30	86.5	52.4	122	121	37.3	35.8	19.4	15.4
Min 2018	4.22	1.61	9.9	3.57	0.30	0.23	70.5	39.0	118	118	23.4	28.2	14.3	10.4
Max 2018	6.56	3.76	17.5	8.88	0.48	0.36	98.0	63.5	129	128	50.5	44.2	26.4	19.6
Mean 2019	7.94	4.37	26.6	18.0	0.43	0.25	84.9	63.4	194	191	33.5	30.7	21.9	15.3
Min 2019	6.16	2.55	21.5	13.0	0.27	0.17	73.0	50.0	186	186	25.1	24.2	17.1	11.1
Max 2019	9.62	5.77	35.0	24.0	0.52	0.31	96.0	74.0	201	196	39.6	38.5	26.8	18.6
**CY Mean**	**6.61**	**3.51**	**19.7**	**12.1**	**0.36**	**0.34**	**83.7**	**57.3**	**182**	**180**	**36.6**	**32.1**	**17.4**	**18.6**
CY Min	5.25	2.48	16.8	9.2	0.28	0.23	72.1	46.8	176	176	26.6	25.7	14.5	14.8
CY Max	7.81	4.37	23.8	15.9	0.41	0.40	94.6	68.2	188	186	45.7	37.7	21.4	22.2
	LSD		LSD		LSD		LSD		LSD		LSD		LSD	
G (Genotype)	0.70	***	2.95	**	0.04	***	4.20	***	1.40	***	2.81	***	1.86	***
T (Treatment)	1.47	**	2.9	**	0.01	*	7.60	**	0.50	***	3.84	*	1.80	
Y (Year)	0.60	***	2.24	***	0.05	**	5.50	*	1.50	***	1.27	*	1.25	***
T*G	1.27		4.35		0.06		7.10	*	1.90	**	4.38	*	2.74	**
Y*G	1.02	***	4.25		0.06	*	6.50		2.10	***	3.98	***	2.67	***

Grain yield (GY t ha^-1^), above ground dry matter (AGDM t ha^-1^), harvest index (HI), plant height (PH cm), days to heading (HD), thousand grain weight (TGW), and fruiting efficiency (FE grains g^-1^).

Significance levels displayed as ns > .05, * <.05 >.01, ** <.01, ***<0.001.

**Fig 2:**
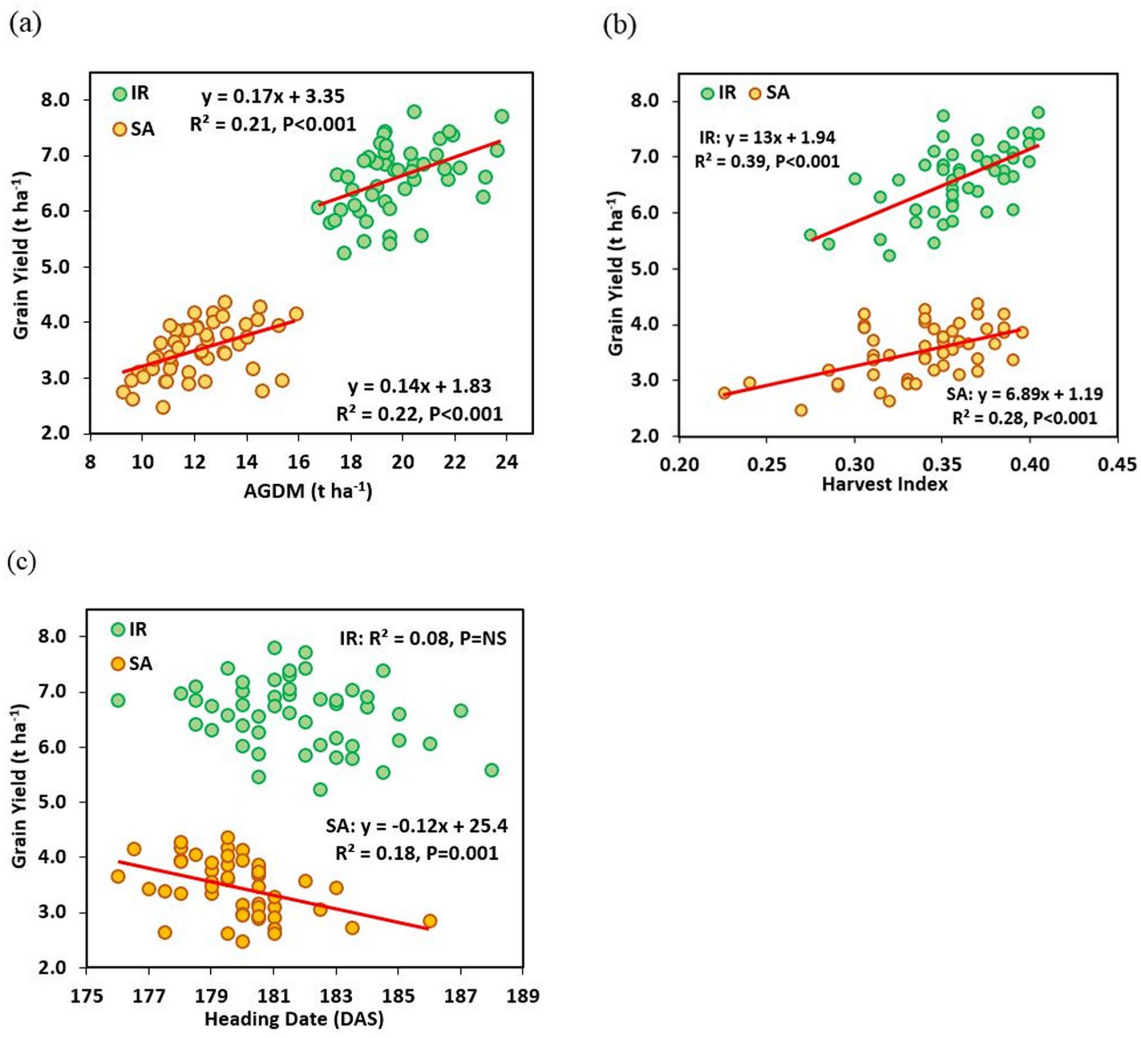
Linear regression amongst 50 wheat genotypes between GY and (a) above ground dry matter (AGDM) (b) harvest index and (c) Heading date (DAS) under irrigated (IR) and semiarid (SA) conditions (mean of 2018 and 2019).

Regression analysis showed a positive correlation amongst the genotypes for GY between IR and SA conditions (R^2^ = 0.27, *P*<0.001, Fig 1a). Nevertheless, some genotypes marked differences in percentage reduction in GY; for example: genotype 33 with a 49.3% reduction compared to genotypes 16 and 25 with 34.8 and 40.7% reduction, respectively, under SA conditions (S2 Table). Overall, genotypes 39, 18 and 29 showed relative less yield reduction under SA conditions indicating ability to tolerate the drought whereas genotype 40, 35 and 29 were the most susceptible to drought. GY also showed positive correlation with AGDM (IR: R^2^ = 0.21, *P*<0.001 and SA: R^2^ = 0.22., *P*<0.001) and NDVI at anthesis (IR: R^2^ = 0.18, *P* = 0.01 and SA: R^2^ = 0.23., *P*<0.001) under both IR and SA conditions (Fig 2a and 2c).

For harvest traits, averaging across years, overall the AGDM was the component affected most by the semiarid conditions reducing from 19.7 to 12.1 t ha^-1^ (-38.6%, *P* = 0.01, Table 1); whereas TGW reduced from 36.6 to 32.1 (-12.2%, *P* = 0.03, Table 1). For AGDM, T x G interaction was not significant whereas for TGW it was (*P* = 0.03). Reduction in grains per ear under SA conditions ranged from -0.5% (genotype 16) to -49.0% (genotype 9). Variation in response to drought for TGW was from -0.9 to -25.7%. Heading date (HD) was advanced by two days in SA conditions compared to IR (*P* = 0.004). There was a negative association between GY and HD amongst cultivars under SA conditions (R^2^ = 0.18, *P* = 0.002), but no association under IR conditions (Fig 2c). Overall drought reduced plant height by 31.6% but there was no correlation between GY and PH amongst cultivars under either SA or IR conditions. Harvest index showed a positive correlation with GY under IR (R^2^ = 0.39, *P*<0.001) and SA (R^2^ = 0.28, *P*<0.001) conditions (Fig 2b).

### 3.2 Root system traits and correlations with yield and yield components

In 2018, root traits were not significantly affected by the irrigation treatment. However, differences between the genotypes were observed in all the root traits (*P*<0.05, Table 2). Overall root surface area ranged from 28.4 to 59.4 cm^2^ per plant with mean of 44.3 cm^2^ per plant and 22.9 to 60.1 cm^2^ per plant with mean of 43.4 cm^2^ per plant under IR and SA conditions respectively. Interestingly under SA conditions, GY and AGDM showed negative correlation with root surface area (r = -0.29 and r = -0.32), and root volume (r = -0.26 and r = -0.28) per plant. However, TGW showed positive association with root surface area (r = 0.40), root diameter (r = 0.52), and root volume (r = 0.49). Under IR conditions root diameter and TGW showed positive correlation with GY (r = 0.27, and r = 0.29 respectively). Also, under IR conditions onset of canopy senescence showed a positive correlation with root diameter (r = 0.29, Table 4).

**Table 2:** ANOVA showing significance for genotype (G), treatment (T), interaction (G x T) and genetic ranges for root and senescence traits: Root surface area (RoSuAr), root diameter (RoDiM), root volume (RoVol), NDVI at anthesis (NDVI), senescence start (SenSt), senescence duration (SenDu) in 2018.

	RoSuAr (cm2)		RoDiM (mm)		RoVol (cm^-3^)		NDVI		SenSt (°CD)		SenDu (°CD)	
	IR	SA	IR	SA	IR	SA	IR	SA	IR	SA	IR	SA
Mean	44.3	43.4	0.52	0.51	0.57	0.56	0.63	0.52	648	250	1001	1501
Min	28.4	22.9	0.42	0.41	0.31	0.24	0.56	0.40	425	149	669	1131
Max	59.4	60.1	0.69	0.64	0.86	0.87	0.71	0.61	1033	461	1401	2221
%red		2.17		1.36		2.82		17.6		61.4		-49.9
**LSD**												
G	11.6	***	0.08	***	0.19	***	0.56	***	127	***	243	***
T	32.6		0.25		0.13		1.45	T	114	**	8.43	***
T x G	17.13		0.12		0.26		0.82	*	179	***	341	***

Significance levels displayed as ns>.10, T <.10 & > .05, * <.05 >.01, ** <.01, ***<0.001.

In 2019, root diameter (RoDiM), root number per plant (RoNoPl) and root:shoot ratio (Ro:Sh ratio) showed significant differences between genotypes and treatments (Table 3). Overall phenotypic variation amongst genotypes for root angle was from 46.7° to 68.0° with mean of 56.6° and 46.1° to 63.8° with mean of 56.3° under IR and SA conditions, respectively. Root number per shoot (RoNoSh) showed a positive correlation with GY and HI (r = 0.32 and r = 0.35, respectively) under SA conditions but there was no correlation under IR conditions (Table 4). AGDM showed a negative correlation with root diameter (r = -0.29) and root dry weight per plant (r = -0.49) under IR conditions. Wider root angle was also correlated with higher GY and AGDM under IR conditions (r = 0.29 and r = 0.33, respectively; Table 5). Narrower root angle was correlated with more roots per plant under SA conditions, whereas under IR conditions these correlations were not significant. Root dry weight per plant also showed a positive correlation with root diameter under IR conditions. There was a strong positive correlation between root dry weight and root number per plant under both SA and IR conditions.

**Table 3:** ANOVA showing significance for genotype (G), treatment (T), interaction (G x T) and genetic ranges for root and senescence traits: Root angle (RoAng), root diameter (RoDiM), root dry weight per plant (RoDrWtPl), root number per plants (RoNoPl), root:shoot ratio (Ro:Sh Ratio), canopy temperature (CT), canopy green area per meter square at anthesis (GA) and NDVI at anthesis (NDVI) studied in 2019.

	RoAng (°)		RoDiM (mm)		RoDrWtPl (g)		RoNoPl		Ro:Sh Ratio		CT (°C)		GA		NDVI	
	IR	SA	IR	SA	IR	SA	IR	SA	IR	SA	IR	SA	IR	SA	IR	SA
Mean	56.6	56.3	0.71	0.68	0.52	0.49	19.9	15.2	0.08	0.06	29.3	36.9	1.94	1.68	0.72	0.57
Min	46.7	46.1	0.46	0.50	0.15	0.20	13.8	10.8	0.03	0.02	26.0	31.5	1.82	1.43	0.64	0.46
Max	68.0	63.8	0.87	0.79	0.99	0.99	27.0	23.3	0.17	0.10	32.0	43.0	2.00	1.90	0.79	0.66
%red		0.51		4.54		6.62		23.9		27.6		-26.2		13.1		20.8
**LSD**																
G	9.09		0.13	*	0.32		5.28	*	0.04	T	4.19		0.13	**	0.06	***
T	1.82		0.03	**	0.06		1.06	***	0.01	***	0.84	***	0.02	***	0.01	***
T X G	12.9		0.18		0.45		7.47		0.05		5.93		0.19		0.08	*

Significance levels displayed as ns>.10, T <.10 & > .05, * <.05 >.01, ** <.01, ***<0.001.

**Table 4:** Correlation matrix showing correlation coefficient (r) values for grain yield (GY), above ground dry matter (AGDM), harvest index (HI), thousand grain weight (TGW), heading date (HD), root surface area (RoSuAr), root diameter (RoDiM), root volume (RoVol), NDVI at anthesis (NDVI), NDVI senescence start (SenSt), NDVI senescence duration (SenDu). Below diagonal IR and above diagonal SA for 2018.

	**GY**		**AGDM**		**HI**		**TGW**		**HD**		**RoSuAr**		**RoDiM**		**RoVol**		**NDVI**		**SenSt**		**SenDu**	
**GY**	-	-	0.87	***	0.51	***	0.04		-0.35	**	-0.29	*	-0.08		-0.26	T	0.34	**	0.14		-0.10	
**AGDM**	0.72	***	-	-	0.03		-0.02		-0.30	*	-0.32	*	-0.10		-0.28	*	0.23		0.03		0.02	
**HI**	0.21		-0.51	***	-	-	0.09		-0.25	T	-0.05		0.00		-0.05		0.27	T	0.26	T	-0.25	T
**TGW**	0.29	*	-0.01		0.40	**	-	-	-0.18		0.40	**	0.52	***	0.49	***	0.01		0.01		0.07	
**HD**	-0.11		0.18		-0.38	**	-0.28	*	-	-	0.20		-0.14		0.12		0.10		-0.14		-0.14	
**RoSuAr**	0.02		0.03		0.02		0.23		0.31	*	-	-	0.37	**	0.91	***	-0.06		-0.07		0.11	
**RoDi**	0.27	*	0.11		0.16		0.43	***	-0.23		0.16		-	-	0.68	***	-0.12		0.03		0.04	
**RoVo**	0.16		0.09		0.10		0.43	**	0.13		0.84	***	0.65	***	-	-	-0.09		-0.03		0.09	
**NDVI**	0.34	**	0.36	**	-0.07		-0.03		0.26	T	-0.03		-0.14		-0.08		-	-	0.48	***	-0.63	***
**SenSt**	0.38	**	0.30	*	0.03		0.35	**	-0.33	*	0.08		0.29	*	0.20		-0.16		-	-	-0.76	***
**SenDu**	-0.27	*	-0.17		-0.05		-0.25	T	0.11		-0.16		-0.26	T	-0.25	T	0.13		-0.74	***	-	-

Significance levels displayed as ns>.10, <.10 T > .05, * <.05 >.01, ** <.01, ***<0.001.

**Table 5:** Correlation matrix showing correlation coefficient (r) values for grain yield (GY), above ground dry matter (AGDM), harvest index (HI), thousand grain weight (TGW), days to heading (DH), canopy green area per meter square at anthesis (GA An) and after 2 weeks of anthesis (GA 2W), NDVI at anthesis (NDVI), root angle (RoAng), root diameter (RoDiM), root dry weight per plant (RoDrWtPl), root number per shoot (RoNoSht), and canopy temperature at anthesis (CT). Below diagonal IR and above diagonal SA for 2019.

	**GY**		**AGDM**		**HI**		**TGW**		**HD**		**GA An**		**GA 2W**		**NDVI**		**RoAng**		**RoDiM**		**RoDrWtPl**		**RoNoSht**		**CT**	
**GY**	-	-	0.34	*	0.62	***	0.51	***	-0.40	**	0.56	***	0.76	***	0.55	***	0.11		-0.06		-0.01		0.32	*	-0.28	*
**AGDM**	0.43	**	-	-	-0.51	***	-0.08		-0.18		0.04		0.10		0.01		0.10		0.02		-0.07		-0.06		-0.15	
**HI**	0.37	**	-0.60	***	-	-	0.55	***	-0.22		0.44	**	0.59	***	0.47	***	0.00		-0.07		0.06		0.35	**	-0.15	
**TGW**	0.16		-0.20		0.22		-	-	-0.31	*	0.16		0.40	**	0.25	T	0.06		0.05		0.02		0.27	T	-0.15	
**HD**	-0.33	*	-0.34	*	0.17		-0.12		-	-	0.12		0.05		0.01		-0.04		-0.21		0.06		-0.06		-0.07	
**GA An**	0.36	**	-0.01		0.42	**	-0.25	T	0.26	T	-	-	0.74	***	0.86	***	-0.12		-0.12		0.02		0.10		-0.39	**
**GA 2W**	0.25	T	0.08		0.28	*	-0.17		0.27	*	0.89	***	-	-	071	***	0.15		-0.04		0.02		0.35	**	-0.42	**
**NDVI**	0.26	T	0.02		0.36	**	-0.18		0.39	**	0.69	***	0.74	***	-	-	-0.02		-0.08		-0.05		0.03		-0.31	*
**RoAng**	0.29	*	0.33	*	-0.08		-0.15		-0.02		-0.01		-0.09		0.27	T	-	-	-0.08		-0.41	**	-0.01		-0.12	
**RoDiM**	-0.11		-0.29	*	0.11		0.20		0.18		-0.04		0.08		0.18		0.05		-	-	0.04		0.16		0.00	
**RoDrWtPl**	-0.19		-0.49	***	0.35	*	0.26	T	0.27	T	0.08		0.20		0.13		-0.25	T	0.34	*	-	-	-0.06		-0.08	
**RoNoSht**	-0.15		0.06		-0.15		-0.05		0.33	*	-0.03		-0.13		-0.02		-0.04		0.10		-0.07		-	-	-0.18	
**CT**	0.07		0.00		-0.07		0.11		-0.17		-0.27	T	-0.21		-0.17		0.10		0.18		-0.18		0.00		-	-

Significance levels displayed as ns>.10, <.10 T > .05, * < .05 >.01, ** <.01, ***<0.001.

Traits correlations were also explored in a subset of 30 genotypes with median flowering dates and which differed in flowering date by only 1 day in each of the two seasons. The correlation matrices for this group of genotypes are shown in S4 and S5 Tables. However, there was no meaningful change in the correlations between the group of genotypes with narrow flowering date (S4 and S5 Tables) as compared to the correlations for the full set of genotypes.

### 3.3 Canopy senescence and temperature traits

Overall, in 2018 NDVI at anthesis (NDVI, GS61) ranged from 0.56 to 0.71 and 0.40 to 0.61 under IR and SA conditions, respectively. ANOVA shows that there was a significant difference between genotypes and irrigation treatments along with significant G x T interaction for NDVI at anthesis, senescence start (SenSt) and senescence duration (SenDu) (Table 2). There was a positive correlation between NDVI at anthesis and GY under both, IR (r = 0.34) and SA (r = 0.34) conditions (Table 4). Under IR conditions, NDVI at anthesis and senescence start (SenSt) showed positive correlations with GY (r = 0.34 and r = 0.38, respectively) whereas senescence duration (SenDu) showed negative correlation with GY (r = -0.27) (Table 4).

**Fig 3:**
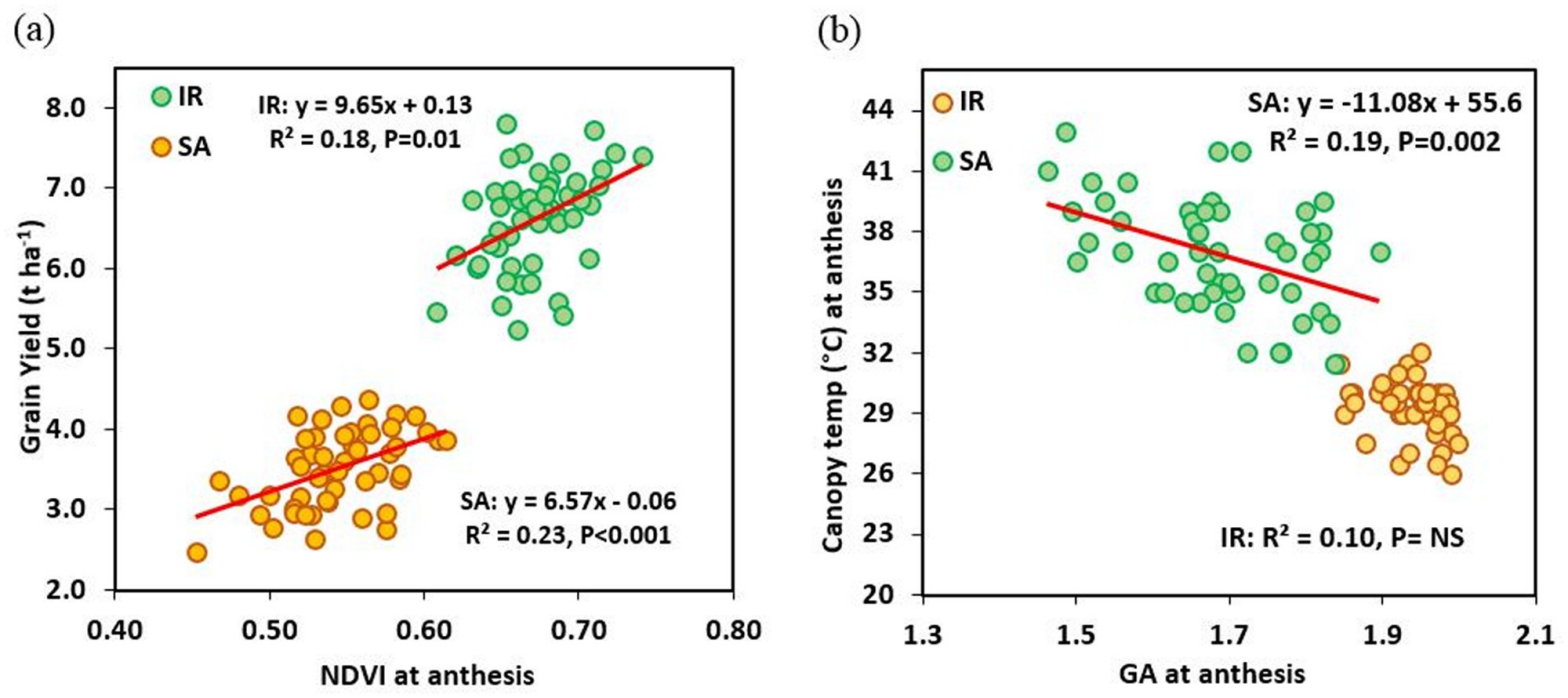
Linear regression amongst 50 wheat genotypes between GY and (a) NDVI at anthesis (b) Green area per meter square (GA) at anthesis under irrigated (IR) and semiarid (SA) conditions (mean of 2018 and 2019) at anthesis in 2019.

In 2019, NDVI at anthesis ranged from 0.64 to 0.79 with mean of 0.72 and 0.46 to 0.66 with mean of 0.57 under IR and SA conditions, respectively. There was significant difference between genotypes and treatments for canopy green area per meter square at anthesis and NDVI at anthesis. G x T interaction was significant only for NDVI at anthesis (Table 3). In terms of correlation between GY and vegetation indexes, canopy green area per meter square at anthesis showed stronger correlation with GY than NDVI at anthesis and these correlations were stronger under SA (r = 0.56 and r = 0.55, respectively) than IR (r = 0.36 and r = 0.26, respectively) conditions (Table 5, Fig 3). Under SA conditions both canopy green area at anthesis and NDVI at anthesis showed a negative correlation (r = -0.39 and r = -0.31, respectively) with canopy temperature (Table 5; Fig 3). Canopy green area and NDVI after two and three weeks of anthesis was not correlated with GY.

### 3.4 Correlation between different yield components under IR and SA condition

Under IR conditions, the first principal component (PC1) explained 25.3% variation and the group of traits explaining this variation were fruiting efficiency, spikelet’s per ear, days to heading with positive effect whereas TGW showed a negative effect. The second principal component (PC2) explained 20.8% variation and the group of traits explaining this variation included grains per ear, harvest index, grain yield, and root diameter with positive effects whereas above ground dry matter showed a negative effect.

**Fig 4:**
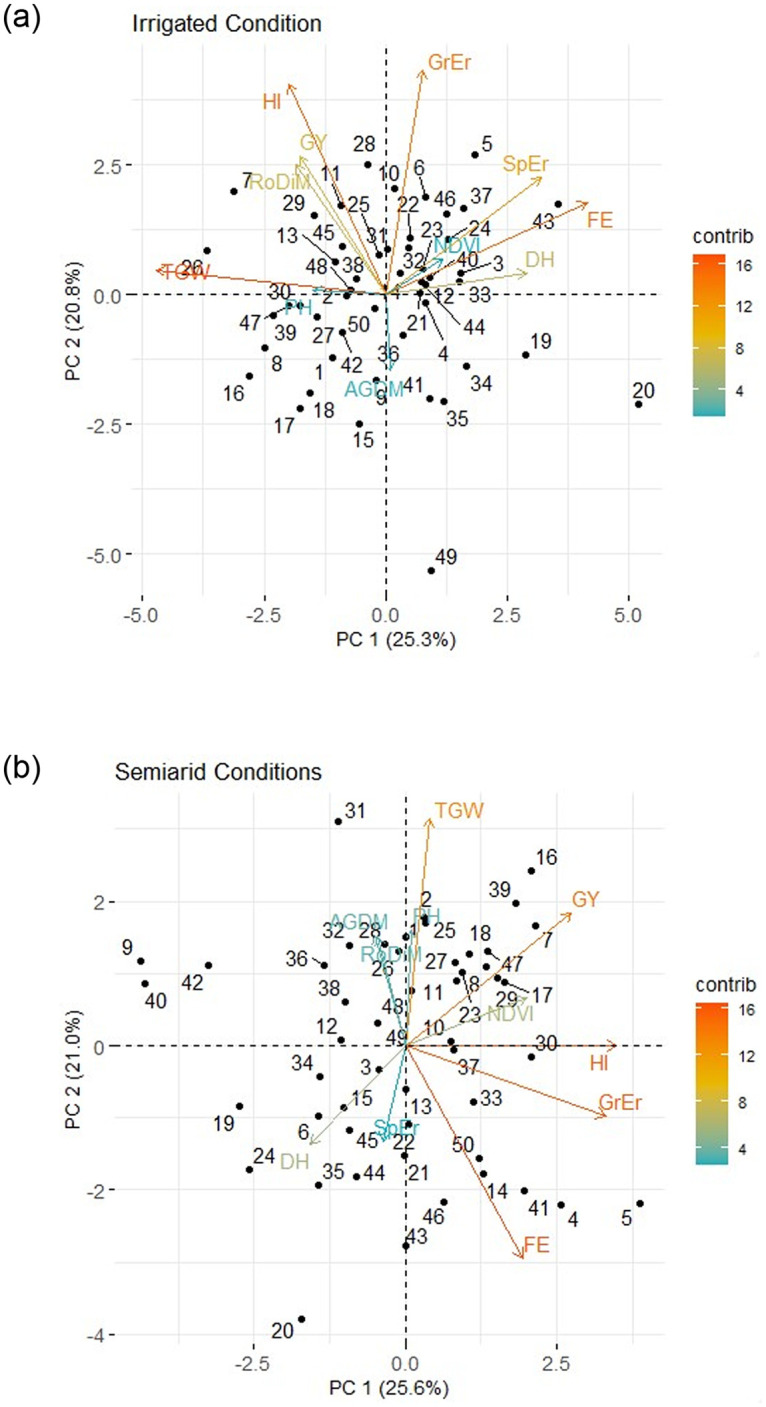
Bi-plot for grain yield (GY), above ground dry matter (AGDM), harvest index (HI), thousand grain weight (TGW), days to heading (DH), grain numbers per ear (GrEr), spikelet number per ear (SpEr), fruiting efficiency at harvest (FE), NDVI at anthesis (NDVI) and root diameter (RoDiM) under irrigated and semiarid conditions for 50 cultivars (Mean of 2018 and 2019). (Contrib: contribution in total variation in per cent–red to blue: stronger to low).

Under SA conditions, PC1 explained 25.6% variation and traits correlated with positive effect—were grains per ear, harvest index, grain yield, and NDVI whereas days to heading showed a negative effect. PC2 explained 21.0% of variation and traits showing correlation with positive effects were—thousand grain weight, plant height, root diameter, whereas spikelets per ear showed a negative effect (Fig 4).

Overall the bi-plots showed genotype code numbers (S1 Table) 11, 28, 7, and 29 under IR and 16, 39, 7 and 18 under SA conditions performed relatively better for GY than other genotypes (Fig 4). There was no grouping of genotypes that indicated strong genotype outliers for correlations with PC1 or PC2 under both treatments.

### 3.5 Stepwise regression analysis

A forward stepwise multiple-linear regression analysis was done to investigate which traits amongst root and shoot traits contributing the most towards improvement in the GY in the SA treatment (S6 Table). The stepwise regression analysis identified NDVI at anthesis (NDVI), root surface area (RoSuAr), and NDVI senescence duration (SenDu) in 2018 and green area at anthesis (GA An), root numbers per shoot (RoNoSh), and root angle (RoAng) in 2019 were the most important traits under drought conditions. These traits explained 17 and 41% of phenotypic variation contributing to GY in 2018 and 2019 respectively and the regression model was not improved by the addition of any further traits.

### 3.6 Marker-traits associations

Marker, allele/haplotype effect, mean of traits for each allele and probability of a significant difference in the mean under IR and SA conditions are presented in Table 6 for 2018 and 2019. In 2018, results showed that marker *TaCwi-4A* had a significant influence on GY under SA. This marker was responsible for increase in GY by 17.1% under SA in the presence of the Hap-4A-C allele which was associated with higher yield under drought conditions [37] and present in 59% of genotypes. Marker *Dreb1* (Hap-I) was responsible for increase root surface area (RoSuAr) and root volume (RoVol) only under SA conditions. *Dreb1* allele *TaDreb-B1a* increased these traits by 9.4% and 13.3%, respectively, under SA conditions. *Dreb1* (Hap-I) was also correlated with extended SenEnd under SA conditions. The *PRR73-A1* gene had a significant influence on GY under SA but not under IR conditions. There was an increase of 14.1% under SA conditions in presence of Hap-II allele which was present in 29% of genotypes studied. This allele was also correlated with extended senescence end (SenEnd) under SA and IR conditions.

**Table 6:** The key markers and haplotype mean for traits: Grain yield (GY t ha^-1^), harvest index (HI), plant height (PH cm), thousand grain weight (TGW g), heading date (HD DAS), root surface area (RoSuAr cm^2^), root diameter (RoDiM mm), root volume (RoVol cm^3^), NDVI senescence end (SenEnd °CD), NDVI Mid senescence (MidSen °CD), canopy green area per meter square at anthesis (GA m2), root angle (RoAng °), root dry weight per plant (RoDrWtPl g), root shoot ratio (Ro:Sh ratio), and canopy temperature (CT °C) under SA and IR conditions along with significant difference between the two haplotype mean to show marker-trait association in 2018 and 2019 for 50 genotypes.

		PRR73.A1				TEF.7A				TaCwi.4A				Dreb1				GY.QTL			
	Hap-I	Early flowering				Low TGW				Drought tolerant				Drought tolerant				High yield			
	Hap-II	Late flowering				High TGW				Drought susceptible				Drought susceptible				Low yield			
		SA		IR		SA		IR		SA		IR		SA		IR		SA		IR	
**2018**																					
GY	Hap-I	2.44	*	5.27	T	2.66	**	5.33		2.75	**	5.41		2.49		5.33		2.40	T	5.29	
	Hap-II	2.84		5.56		2.14		5.35		2.28		5.22		2.61		5.36		2.72		5.39	
TGW	Hap-I	33.3		36.8		33.6		37.4		34.3	T	38.3		33.6		37.9		32.6	*	36.6	
	Hap-II	34.3		38.7		33.2		36.7		32.6		36.2		33.3		36.9		34.5		38.0	
HD	Hap-I	121	T	123	**	121	*	122		121	T	122		121		122		121		122	
	Hap-II	120		121		122		123		122		123		121		122		121		122	
HI	Hap-I	0.42		0.41		0.43		0.42		0.43		0.42		0.41		0.41		0.43		0.42	
	Hap-II	0.44		0.42		0.42		0.42		0.42		0.42		0.44		0.42		0.42		0.41	
PH	Hap-I	51.3	*	85.3	*	53.1	T	86.2		53.7	**	87.2		53.2		88.8	*	50.9	*	85.7	
	Hap-II	55.2		89.6		49.9		87.4		50.1		84.9		51.7		84.8		54.1		87.4	
RoSuAr	Hap-I	43.7		44.7		42.8		43.5		43.5		43.9		45.7	*	45.4		43.3		45.0	
	Hap-II	43.5		43.2		45.5		47.2		43.3		44.4		41.4		43.1		43.4		43.5	
RoDiM	Hap-I	0.50	*	0.51		0.51		0.51		0.51		0.52		0.52		0.52		0.49	**	0.51	
	Hap-II	0.53		0.54		0.52		0.52		0.51		0.51		0.50		0.51		0.53		0.52	
RoVol	Hap-I	0.55		0.57		0.55		0.56		0.57		0.58		0.60	*	0.59		0.55		0.58	
	Hap-II	0.59		0.58		0.60		0.61		0.55		0.56		0.52		0.56		0.57		0.57	
MidSen	Hap-I	920		1338	*	889		1371		910		1383	T	938		1376		988	**	1350	
	Hap-II	871		1412		964		1319		884		1327		907		1350		819		1372	
SenEnd	Hap-I	1698	**	1634	**	1735		1676	*	1729		1675		1763	*	1656		1711		1654	
	Hap-II	1801		1712		1701		1601		1733		1641		1694		1665		1747		1665	
**2019**																					
GY	Hap-I	4.36		7.75		4.46		7.98		4.338		7.91		4.28		7.90		4.29		7.67	T
	Hap-II	4.38		8.14		4.08		7.57		4.446		7.86		4.48		7.91		4.47		8.14	
TGW	Hap-I	30.9		35.4		31.0		36.2		30.9		35.8		30.3		35.5		29.8	T	35.0	
	Hap-II	30.4		36.8		29.7		34.6		30.7		36.0		31.0		36.2		31.7		36.8	
HD	Hap-I	191	*	194	*	191	*	193	*	191	*	193		191		194		191		194	
	Hap-II	190		192		192		196		192		195		191		194		191		193	
HI	Hap-I	0.25		0.30		0.25		0.30		0.254		0.30		0.24	*	0.30		0.25		0.30	
	Hap-II	0.25		0.31		0.25		0.31		0.248		0.31		0.26		0.31		0.25		0.30	
PH	Hap-I	62.9		84.1	T	63.8		85.4		64.0		85.7		65.1		87.1	*	63.9		85.2	
	Hap-II	64.6		87.4		62.7		84.1		62.6		84.1		62.5		83.9		63.1		85.0	
GA	Hap-I	1.69		1.94		1.68		1.94		1.686		1.94		1.65	*	1.94		1.69		1.95	*
	Hap-II	1.67		1.95		1.69		1.95		1.685		1.93		1.71		1.94		1.67		1.93	
RoDiM	Hap-I	0.68		0.73		0.68		0.71		0.674		0.71		0.69		0.70		0.68		0.69	*
	Hap-II	0.69		0.70		0.69		0.73		0.700		0.72		0.68		0.72		0.69		0.74	
RoDrWtPl	Hap-I	0.51		0.54		0.48		0.50	T	0.467		0.49	T	0.52		0.53		0.52		0.55	
	Hap-II	0.46		0.50		0.54		0.62		0.522		0.59		0.46		0.51		0.46		0.49	
Ro:Sh Ratio	Hap-I	0.062	*	0.09		0.06		0.08	*	0.055	*	0.08		0.06		0.09		0.063	T	0.09	
	Hap-II	0.051		0.08		0.06		0.10		0.064		0.09		0.06		0.08		0.054		0.08	
CT	Hap-I	36.9		29.5		37.2		29.7	**	36.9		29.2		36.4		29.2		36.8		29.2	
	Hap-II	37.1		29.1		36.2		28.4		37.1		29.8		37.2		29.5		37.1		29.7	

Significance levels displayed as ns>.10, T <.10 & > .05, * <.05 >.01, ** <.01, ***<0.001.

Note: Frequency: *PRR73*.*A1*—early flowering (35) and late flowering (14), *TEF*.*7A*—lower TGW (39) and high TGW (11), *TaCwi*.*4A*—high yield in drought (29) and low yield in drought (20), *Dreb1*- drought tolerant (20) and drought susceptible (29), *ISBW11*.*GY*.*QTL*.*CANDIDATE* (GY.QTL)—higher yield (27) and lower yield (23).

The marker-trait associations in 2018 were not apparent for the same traits in 2019. However, Marker *PRR73-A1* with allele Hap-I (early flowering) was responsible for increase root: shoot ratio (Ro:Sh Ratio) in 2019.

## 4. Discussion

In this discussion we address the potential application of the physiological traits and molecular markers examined in the field experiments in trait-based breeding for drought tolerance in wheat. We consider first the effect of phenology and canopy traits on the yield responses of genotypes to drought, then the effects of root traits and lastly the association between the molecular markers and responses to drought.

### 4.1 Grain yield responses to drought and association with phenology

In our experiments, comparing the two irrigation treatments, there was a moderately to severe drought with overall 3.06 t ha^-1^ (-47%) yield reduction under SA conditions. This is representative of Mediterranean drought effects in semi-arid conditions reported for wheat with reductions of yield typically ca. 30–50% [45]. The cultivars responded differently to the drought stress as indicated by the significant irrigation x genotype interaction. Higher yield under IR conditions was correlated with greater yield loss under SA conditions amongst the cultivars. From the physiological standpoint, it is not surprising that absolute reduction in yield for a given reduction in water resource is strongly influenced by yield potential. This is because higher yield potential genotypes will tend to use more water during the season and have higher biomass that low yield potential genotypes under optimal conditions [46–48].

Drought had only a small effect on heading date (GS59) advancing on average by one day in 2018 and three days in 2019; indicating genotypes responded similarly to drought stress (Fig 2). In both SA and IR conditions, HD ranged by 10 days amongst cultivars. Correlations between heading date and grain yield were negative under SA conditions in both years, and there was also a negative correlation under IR conditions in 2019 although it was less strong than under SA conditions. Bi-plots for the cross-year means also confirm these effects (Fig 4). This correlation among genotypes did not change even when considering just the median 30 genotype as a narrower flowering date group with a difference of 1 day in their flowering dates (S4 and S5 Tables). Early flowering has been correlated with drought escape in wheat in environments subjected to severe early season drought stress, e.g., in northern Mexico [46]. Similarly, Worland *et*. *al*. [49] reported increased yield for *Ppd-D1a* early-flowering NILs by ca. 5% compared to *Ppd-D1b* controls in dry years. Each day’s advancement in HD raised yield by 0.11 t ha^-1^. In the present study soil depth was more than 2 m at the field site with a very low organic matter. Presumably a shorter pre-anthesis phase reduced water uptake during this phase, so that season-long water uptake was redistributed more favorably with regard to the post-anthesis period; therefore water uptake during grain filling was increased with earlier flowering. However, in one year a similar negative correlation between HD and yield was recorded under irrigated conditions. This indicated that the negative association may have been partly correlated with advanced flowering leading to cooler prevailing temperatures during grain filling and therefore more calendar days for grain filling [50]. Brdar *et*. *al*. [51] showed that during grain filling, an increase in 1°C mean daily temperature higher than optimum can be responsible for decrease in ca. 2.8 mg of grain weight. Observations regarding flowering time and drought resistance are very much dependent on the exact timing of drought stress and we recognize that the present experiments would need to be repeated over more years before we could conclude with certainty that later heading date overall has a negative effect on yield losses under droughts in Turkey. For example, it may be that there is a trade-off between early flowering and the development of a smaller root system, as suggested by Foulkes *et*. *al*. [52].

### 4.2 Correlations between canopy senescence traits and responses to drought

In the present study, greater yield amongst cultivars was correlated with higher green area per meter square and NDVI at around heading or anthesis under both drought and irrigated conditions. This was also confirmed by the stepwise multi-linear regression analysis which identified green area per meter square and NDVI as amongst the most important traits contributing to GY under drought conditions. Both of these are high-throughput measurements. This likely reflected a correlation between NDVI and biomass at anthesis and hence grains m^-2^ under both treatments. Genetic variation in GY in wheat has previously been correlated with green canopy area duration under drought in wheat [47, 53–56], and sorghum [57]. The role of senescence dynamics—start, end and rate of senescence—is important as they relate to grain filling duration and post-anthesis water and N uptake under abiotic stress [56, 58]. Under irrigated conditions, GY was correlated positively with onset of senescence (SenSt) in 2018. Genotypes having delayed onset of senescence may be able to accumulate more plant nutrients and carbohydrates during grain filing duration resulting in higher yield. Our results suggested that there was source limitation if grain growth even under irrigated conditions. This could have been due to some heat stress incurred in the experiments combined with the higher grain number in irrigated conditions leading to source limitation during the later stages of grain filling [59].

One of the objectives of this experiment was to compare the NDVI and RGB-based vegetation indexes as methods for measuring canopy green area. We found that the RGB-based vegetation indexes showed better correlation with GY than NDVI measured with the handheld Trimble GreenSeeker and in addition it increased throughput. Better correlation of grain yield with RGB-based vegetation indexes than NDVI was also reported in durum wheat by Kefauver *et*. *al*. [27] and Fernandez-Gallego *et*. *al*. [28]. The role of a cooler canopy at anthesis correlated with a deeper root system for drought tolerance is previously reported [5] and was also indicated in this experiment with a negative correlation between GA at anthesis, NDVI at anthesis and GY with canopy temperature (CT) under SA conditions in 2019.

### 4.3 Effect of root traits on responses to drought

Shovelomics is a high-throughput phenotyping method for field-grown crops and has been used to quantify genetic variation in root traits in maize [19, 21, 22], legumes [60] and barley [23]. Maccaferri *et*. *al*. [24] carried out field shovelomics for durum wheat recombinant inbred lines (RIL) for crown root length, number and angle and reported QTL. In our study we applied a shovelomics methodology for bread wheat to quantify variation in nodal root angle, length, roots plant^-1^ and roots shoot^-1^ and correlation with GY. In the present study, the range in nodal root angle of 45-65° in the semiarid treatment was similar to that of 42.3–69.2° reported by Maccaferri *et*. *al*. [24] for the Colosseo × Lloyd durum wheat mapping population assessed at anthesis in the field under optimum agronomic conditions in Italy. Values under irrigation of 45.0–70.0° were also similar to those reported by Maccaferri *et*. *al*. [24] under irrigation. The correlation of shallower root angle with higher GY under irrigated conditions in 2019 was possibly correlated with increased root density at shallower depth and increased recovery of fertilizer N uptake which is predominately distributed in the top 30 cm of the soil. Shallow roots may also have increased rate of uptake of irrigation water leading to more transpiration and cooler canopies, so avoiding heat stress. Generally shallower root angle is correlated with shallower root distribution in soil and narrow root angle correlated with relatively deeper root distribution in soil [16]. In durum wheat, Hassouni *et*. *al*. [61] reported 20 to 40% yield advantage under irrigated conditions with the shallow root types compared to deeper root types.

Fan *et*. *al*. [62] reported 46.2% and 68.3% of wheat roots were distributed in the upper 15 and 30 cm, respectively. In contrast, a steeper angle would be expected to correlate with relatively deeper roots and greater yield under SA conditions, as has been reported in wheat in Australia [14–16]. In our experiments soil depth was more than 2 m. Previous studies in maize found steeper root angle related to increased rooting depth under low nitrogen field environments in the USA and South Africa [19]. However, in our results we did not see a significant correlation between root angle and GY under drought. Nevertheless, under SA conditions in 2019 there was a positive correlation between nodal root number per shoot and GY and HI, but no correlation under IR conditions. These results suggest that the wheat ideotype for drought tolerance may be a plant with relatively few tillers but a high number of nodal roots per shoot correlated with a longer residence time per root on average during the season and increased rooting depth. The multi-linear regression model also predicted the best combination of traits for drought-tolerance breeding to include root surface area, root number per shoot and root angle. A field study in Pennsylvania in maize found that reduced nodal roots per plant led to increased root length at depth and 57% higher grain yield under water-stressed conditions [63]. Tillering influences carbon partitioning, and there is some evidence that reduced tillering increases rooting depth in wheat [64, 65] and rice [66].

In 2018 terminal stress was severe as rainfall was lower towards the end of crop season than in 2019 (Table 7). In 2018 under SA conditions genotypes which had less root surface area and root volume per plant produced higher yield and biomass. It is feasible that less surface area and/or volume of surface crown roots may have been correlated with more roots distributed relatively deeper in 2018 under SA conditions. Overall under drought conditions fine roots with smaller root diameter may have an advantage in exploring an increase area of soil relative to the energy invested to grow them [67]. In 2019, when terminal drought was less severe, this correlation with root surface area and volume was not observed.

**Table 7:** Environmental conditions during two field crop growing seasons (2018 and 2019) at experimental site Konya, Turkey.

	2017–18		2018–19	
	Temperature (^○^C) Mean (Min, Max)	Rainfall (mm)	Temperature (^○^C) Mean (Min, Max)	Rainfall (mm)
**Nov**	6.07 (-0.36, 12.5)	70.0	6.52 (0.92, 12.1)	27.4
**Dec**	3.79 (-2.02, 9.6)	18.6	3.29 (-0.47, 7.05)	63.4
**Jan**	1.61 (-2.66, 5.88)	4.60	0.75 (-3.92, 5.44)	66.6
**Feb**	5.93 (-0.35, 12.2)	0.20	4.25 (-0.99, 9.49)	31.6
**Mar**	9.78 (1.86, 17.7)	36.0	6.24 (-0.78, 13.3)	20.8
**Apr**	13.4 (4.95, 21.8)	14.4	9.59 (2.63, 16.5)	32.0
**May**	17.5 (9.45, 25.5)	72.2	17.12 (7.98, 26.2)	10.2
**Jun**	20.8 (12.3, 29.3)	38.8	21.23 (13.4, 29.0)	45.6
**Jul**	24.4 (16.6, 32.2)	20.4	22.76 (15.3, 30.1)	7.60
**Total**		**275.2**		**305.2**

Monthly temperature means, (minimum, maximum) and monthly rainfall.

Shovelomics is becoming an increasingly popular method for the high-throughput phenotyping of roots in field-grown crops. The shovelomics method we have applied for phenotyping nodal root traits in winter wheat in the present study were shown to be a valuable technique. In addition, the negative correlation of canopy temperature with GY indicates that water uptake at depth was contributing to yield increase. As the shovelomics methodology measures root traits in top 20 cm of soil profile, this technique requires further validation that it is a reliable indicator for roots at depth. Shovelomics is a relatively high-throughput method, making it possible in the present study to sample, wash and measure root crowns from 200 plots in two person-days. The shovelomics phenotyping platform is significantly faster than field soil coring, which would take approximately one person-month to sample, wash and extract roots, and image the samples for 200 plots in the present study. This high-throughput shovelomics platform can potentially be used to phenotype large populations to identify QTL, search for candidate genes and develop molecular marker for marker-assisted selection [68]. There are examples of deploying QTL for root depth in other cereal species. In rice, the *Dro1* gene related to steeper crown root angles and deeper rooting was identified by measuring nodal root traits in a high-throughput controlled environment study [69] and has been used to produce drought tolerant NILs which have been phenotyped in field conditions [70].

### 4.4 Association between molecular markers and responses to drought

Genomic studies using high-throughput genotyping assays like KASP have made it possible to genotype large populations at various loci within a very short time [44]. Several recent studies used KASP markers to identify the allelic variation of functional genes in wheat cultivars from China [44], United States [71], and Canada [72]. In our study the clear association of *TEF-7A* and *TaCwi-4A* with GY and *Dreb1* with root surface area (RoSuAr) under SA conditions indicated the usefulness of deploying these markers in wheat breeding for drought tolerance.

Dehydration responsive element binding proteins, *Dreb1*, have been shown to be induced by water stress, low temperature and salinity [73]. In this study, *TaDreb1* was associated with increased root surface area, root volume and delayed end of senescence which indicated multi-trait effects of this transcription factor which were not previously reported. The *TaCwi-4A* marker was correlated with GY, root shot ratio (Ro:Sh ratio) and PH under SA conditions. It was previously reported that storage carbohydrate accumulation in drought susceptible and tolerant cultivars depends on the expression of gene for cell wall invertase (*TaCwi*) in anthers [74]. The effect of drought on pollen fertility is irreversible and may cause grain loss or yield reduction under drought conditions. Since these genes tightly control sink strength and carbohydrate supply, deployment of favorable alleles of these genes could maintain pollen fertility and grain number in wheat. The drought tolerance and correlation of yield-related traits in CIMMYT winter wheat germplasm was strongly associated with *TaCwi-4A* which ultimately increased the grain sink size during drought stress. The selection of favorable allele for *TaCwi-4A* gene can enhance drought adaptability in marker-assisted breeding. Similarly, the association of several important traits like SenEnd, GY, HD, and GA with the flowering time related gene *PRR73-A1* is interesting and indicated the expanded role of this plant development gene in senescence timing. Previously, Zhang *et*. *al*. [36] identified that *PRR73-A1* was associated with plant height and explained up to 7.5% of the total phenotyping variability in Chinese wheats. This gene also showed association with plant height in this experiment in 2018. It was previously observed that flowering time related genes are very important for wheat adaptability in target environments, and these genes are correlated with several yield component traits [38]. Our results provided a set of target genes which could be manipulated to further fine-tune the expression of important drought-tolerance traits.

## 5. Conclusion

In the Mediterranean environment wheat is grown mostly under semiarid conditions and frequent drought affecting wheat yield severely. The strategy of developing drought-tolerant wheat varieties depends on understanding and identifying below-ground and above-ground traits for drought tolerance together with use of marker-assisted selection. In this experiment we used high-throughput root phenotyping techniques like shovelomics for characterizing root system architectural and RGB imaging based vegetation index for canopy senescence dynamic traits. We conclude that higher number of crown roots per shoot was a key trait for yield increase under drought conditions. Use of the RGB-based vegetation index to characterise the canopy green area dynamics could save time and increase precision in selection. The strong correlation of green area index with GY at flowering was encouraging and indicated this index can be used as tool for early stage selection for higher GY. In this study we have evaluated five established functional genes for drought-tolerance traits in field conditions that were previously reported elsewhere and for the first time we have validated them in Turkish wheat breeding lines. The genetic marker *TaCwi*.*4A*, responsible for drought tolerance was associated with higher GY in drought conditions in these experiments and could be used for future breeding. Our results also provided new insight on effects of root system architecture traits, for example, the importance of root angle under irrigated conditions and roots per shoot under drought for increasing grain yield which could be important for developing drought-tolerant cultivars.

## Supporting information

S1 Table Code number, genotype names, genotype origin country, grain yield (t ha^-1^) under IR and SA conditions, % yield reduction, and ranking of yield reduction for 50 advance breeding lines studied at Konya, Turkey in 2018 and 2019 under irrigated and semiarid conditions. (DOCX)

S2 Table Mean, maximum, minimum, LSD for grain yield (GY), percent GY reduction under SA (%Red), above ground dry matter (AGDM), thousand grain weight (TKW), harvest index (HI), plant height (PH), heading date (HD), ear length (EarL), spikelets per ear (Spkt Ear^-1^), fruiting efficiency at harvest (FE), NDVI at anthesis (NDVI) for 50 genotypes under IR and SA conditions (mean of 2018 and 2019). (DOCX)

S3 Table Traits definition and a list of traits measured in 2019, which section of a crown measured from, and their derivations. (DOCX)

S4 Table Correlation matrix showing correlation coefficient (r) values and *P*-values (in orange color) for grain yield (GY), above ground dry matter (AGDM), harvest index (HI), thousand grain weight (TGW), heading date (HD), root surface area (RoSuAr), root diameter (RoDiM), root volume (RoVol), NDVI at anthesis (NDVI), NDVI senescence start (SenSt), NDVI senescence duration (SenDu) for selected 30 genotypes with one day difference in flowering date under irrigated (IR) and semiarid (SA) conditions for 2018. (DOCX)

S5 Table Correlation matrix showing correlation coefficient (r) values and *P*-values (in orange color) for grain yield (GY), above ground dry matter (AGDM), harvest index (HI), thousand grain weight (TGW), days to heading (DH), canopy green area per meter square at anthesis (GA An) and after 2 weeks of anthesis (GA 2W), NDVI at anthesis (NDVI), root angle (RoAng), root diameter (RoDiM), root dry weight per plant (RoDrWtPl), and canopy temperature at anthesis (CT) for selected 30 genotypes with one day difference in flowering date under irrigated (IR) and semiarid (SA) conditions for 2019. (DOCX)

S6 Table Percent yield reduction under SA conditions for 50 genotypes (Mean of 2018 and 2019). Stepwise multi-linear regression with grain yield (GY) as the dependent variable showing the best model for selected traits in irrigated (IR) and semiarid (SA) conditions in 2018 and 2019: Grain yield (GY), root surface area (RoSuAr), root diameter (RoDiM), root volume (RoVol), NDVI at anthesis (NDVI), NDVI senescence start (SenSt), NDVI senescence duration (SenDu) in 2018 and root angle (RoAng), root diameter (RoDiM), root dry weight per shoot (RoDrWtSh), canopy green area per meter square at anthesis (GA An) and NDVI at anthesis (NDVI) in 2019. Independent variables selected in the analyses contributed significantly to the models.
(DOCX)

S1 Fig An example image of the entire wheat root crown showing genetic variation in root system, rectangular scale (top left), and plot ID tag (top right). Some of the measured features include stem width (top green line), system width (bottom pink line), number of nodal roots (middle blue points), and distance from root origin to system width lines (vertical blue line).
(TIF)

## References

[1] Le Treut H. Historical overview of climate change. Climate Change 2007: The Physical Science Basis. Contribution of Working Group I to the Fourth Assessment Report of the Intergovernmental Panel on Climate Change. Cambridge University Press; 2007.

[2] Trnka M, Feng S, Semenov MA, Olesen JE, Kersebaum KC, Rötter RP, et al. Mitigation efforts will not fully alleviate the increase in water scarcity occurrence probability in wheat-producing areas. Sci Adv [Internet]. 2019 9 1;5(9):eaau2406. Available from: http://advances.sciencemag.org/content/5/9/eaau2406.abstract 31579815 10.1126/sciadv.aau2406 PMC6760931

[3] Leng G, Hall J. Crop yield sensitivity of global major agricultural countries to droughts and the projected changes in the future. Sci Total Environ [Internet]. 2019;654:811–21. Available from: http://www.sciencedirect.com/science/article/pii/S0048969718343341 30448671 10.1016/j.scitotenv.2018.10.434 PMC6341212

[4] Reynolds M, Dreccer F, Trethowan R. Drought-adaptive traits derived from wheat wild relatives and landraces. J Exp Bot. 2007;58(2):177–86. 10.1093/jxb/erl250 17185737

[5] Lopes MS, Reynolds MP. Partitioning of assimilates to deeper roots is associated with cooler canopies and increased yield under drought in wheat. Funct Plant Biol [Internet]. 2010;37(2):147–56. Available from: 10.1071/FP09121

[6] Li X, Ingvordsen CH, Weiss M, Rebetzke GJ, Condon AG, James RA, et al. Deeper roots associated with cooler canopies, higher normalized difference vegetation index, and greater yield in three wheat populations grown on stored soil water. J Exp Bot. 2019;70(18):4963–74. 10.1093/jxb/erz232 31089708 PMC6760272

[7] Foulkes MJ, Hawkesford MJ, Barraclough PB, Holdsworth MJ, Kerr S, Kightley S, et al. Identifying traits to improve the nitrogen economy of wheat: Recent advances and future prospects. F Crop Res. 2009 12 12;114(3):329–42.

[8] Lopes MS, Reynolds MP. Stay-green in spring wheat can be determined by spectral reflectance measurements (normalized difference vegetation index) independently from phenology. J Exp Bot. 2012;63(10):3789–98. 10.1093/jxb/ers071 22412185 PMC3388823

[9] Gregory PJ, McGowan M, Biscoe PV, Hunter B. Water relations of winter wheat: 1. Growth of the root system. J Agric Sci. 1978;91(1):91–102.

[10] Wasson AP, Richards RA, Chatrath R, Misra SC, Prasad SVS, Rebetzke GJ, et al. Traits and selection strategies to improve root systems and water uptake in water-limited wheat crops. J Exp Bot. 2012;63(9):3485–98. 10.1093/jxb/ers111 22553286

[11] Lynch JP. Steep, cheap and deep: an ideotype to optimize water and N acquisition by maize root systems. Ann Bot. 2013;112(2):347–57. 10.1093/aob/mcs293 23328767 PMC3698384

[12] Richard CAI, Hickey LT, Fletcher S, Jennings R, Chenu K, Christopher JT. High-throughput phenotyping of seminal root traits in wheat. Plant Methods. 2015;11(1):1–11. 10.1186/s13007-015-0043-0 25750658 PMC4351910

[13] van Noordwijk M. Functional interpretation of root densities in the field for nutrient and water uptake. Instituut voor Bodemvruchtbaarheid; 1983.

[14] Olivares-Villegas JJ, Reynolds MP, McDonald GK. Drought-adaptive attributes in the Seri/Babax hexaploid wheat population. Funct Plant Biol. 2007;34(3):189–203. 10.1071/FP06148 32689345

[15] Manschadi AM, Hammer GL, Christopher JT, Devoil P. Genotypic variation in seedling root architectural traits and implications for drought adaptation in wheat (Triticum aestivum L.). Plant Soil. 2008;303(1–2):115–29.

[16] Manschadi AM, Christopher JT, Hammer GL, Devoil P. Experimental and modelling studies of drought‐adaptive root architectural traits in wheat (Triticum aestivum L.). Plant Biosyst. 2010;144(2):458–62.

[17] Fiorani F, Schurr U. Future scenarios for plant phenotyping. Annu Rev Plant Biol. 2013;64:267–91. 10.1146/annurev-arplant-050312-120137 23451789

[18] Köpke U. Ein Vergleich von Feldmethoden zur Bestimmung des Wurzelwachstums landwirtschaftlicher Kulturpflanzen. Doctoral thesis, Univ. Göttingen. 1979.

[19] Trachsel S, Kaeppler SM, Brown KM, Lynch JP. Shovelomics: high throughput phenotyping of maize (Zea mays L.) root architecture in the field. Plant Soil. 2011;341(1–2):75–87.

[20] Hodgkinson L, Dodd IC, Binley A, Ashton RW, White RP, Watts CW, et al. Root growth in field-grown winter wheat: some effects of soil conditions, season and genotype. Eur J Agron. 2017;91:74–83. 10.1016/j.eja.2017.09.014 29129966 PMC5669304

[21] Lynch JP. Root phenes for enhanced soil exploration and phosphorus acquisition: tools for future crops. Plant Physiol. 2011;156(3):1041–9. 10.1104/pp.111.175414 21610180 PMC3135935

[22] Abiven S, Hund A, Martinsen V, Cornelissen G. Biochar amendment increases maize root surface areas and branching: a shovelomics study in Zambia. Plant Soil. 2015;395(1–2):45–55.

[23] Wojciechowski T, Putz A, Schurr U, Federau J, Fiorani F, Briese C, et al. Root phenotyping of temperate cereals–a high throughput phenotyping pipeline for field experiments. In: International Society of Root Research Symposium. Pflanzenwissenschaften; 2015.

[24] Maccaferri M, El-Feki W, Nazemi G, Salvi S, Canè MA, Colalongo MC, et al. Prioritizing quantitative trait loci for root system architecture in tetraploid wheat. J Exp Bot. 2016;67(4):1161–78. 10.1093/jxb/erw039 26880749 PMC4753857

[25] Tucker CJ. Red and photographic infrared linear combinations for monitoring vegetation. Remote Sens Environ. 1979;8(2):127–50.

[26] Rouse Jr JW. Monitoring the vernal advancement and retrogradation (green wave effect) of natural vegetation. 1973

[27] Kefauver SC, El-Haddad G, Vergara O, Araus JL, Kefauver SC, Vergara-Diaz O, et al. RGB picture vegetation indexes for High-Throughput Phenotyping Platforms (HTPPs). 2015

[28] Fernandez-Gallego JA, Kefauver SC, Vatter T, Gutiérrez NA, Nieto-Taladriz MT, Araus JL. Low-cost assessment of grain yield in durum wheat using RGB images. Eur J Agron. 2019;105:146–56.

[29] Thomas H, Howarth CJ. Five ways to stay green. J Exp Bot. 2000;51(suppl_1):329–37. 10.1093/jexbot/51.suppl_1.329 10938840

[30] Vijayalakshmi K, Fritz AK, Paulsen GM, Bai G, Pandravada S, Gill BS. Modeling and mapping QTL for senescence-related traits in winter wheat under high temperature. Mol Breed. 2010;26(2):163–75.

[31] Todkar L, Harikrishna GP, Jain N, Singh PK, Prabhu K V. Introgression of drought tolerance QTLs through marker assisted backcross breeding in wheat (Triticum aestivum L.). Indian J Genet. 2020;80(2):209–12.

[32] Gautam T, Saripalli G, Kumar A, Gahlaut V, Gadekar DA, Oak M, et al. Introgression of a drought insensitive grain yield QTL for improvement of four Indian bread wheat cultivars using marker assisted breeding without background selection. J Plant Biochem Biotechnol. 2020;1–12.

[33] Liu Q, Zhao N, Yamaguch-Shinozaki K, Shinozaki K. Regulatory role of DREB transcription factors in plant drought, salt and cold tolerance. Chinese Sci Bull [Internet]. 2000;45(11):970–5. Available from: 10.1007/BF02884972

[34] Zheng J, Liu H, Wang Y, Wang L, Chang X, Jing R, et al. TEF-7A, a transcript elongation factor gene, influences yield-related traits in bread wheat (Triticum aestivum L.). J Exp Bot [Internet]. 2014 10 1;65(18):5351–65. Available from: 10.1093/jxb/eru306 25056774 PMC4157721

[35] Wang H, Wang S, Chang X, Hao C, Sun D, Jing R. Identification of TaPPH-7A haplotypes and development of a molecular marker associated with important agronomic traits in common wheat. BMC Plant Biol [Internet]. 2019;19(1):296. Available from: 10.1186/s12870-019-1901-0 31286893 PMC6615193

[36] Zhang W, Zhao G, Gao L, Kong X, Guo Z, Wu B, et al. Functional Studies of Heading Date-Related Gene TaPRR73, a Paralog of Ppd1 in Common Wheat. Front Plant Sci [Internet]. 2016 6 1;7:772. Available from: https://pubmed.ncbi.nlm.nih.gov/27313595 27313595 10.3389/fpls.2016.00772 PMC4887500

[37] Ma D, Yan J, He Z, Wu L, Xia X. Characterization of a cell wall invertase gene TaCwi-A1 on common wheat chromosome 2A and development of functional markers. Mol Breed [Internet]. 2012;29(1):43–52. Available from: 10.1007/s11032-010-9524-z

[38] Khalid M, Afzal F, Gul A, Amir R, Subhani A, Ahmed Z, et al. Molecular characterization of 87 functional genes in wheat diversity panel and their association with phenotypes under well-watered and water-limited conditions. Front Plant Sci. 2019;10:717. 10.3389/fpls.2019.00717 31214230 PMC6558208

[39] Zadoks JC, Chang TT, Konzak CF. A decimal code for the growth stages of cereals. Weed Res [Internet]. 1974 12 1;14(6):415–21. Available from: 10.1111/j.1365-3180.1974.tb01084.x

[40] York LM, Lynch JP. Intensive field phenotyping of maize (Zea mays L.) root crowns identifies phenes and phene integration associated with plant growth and nitrogen acquisition. J Exp Bot. 2015;66(18):5493–505. 10.1093/jxb/erv241 26041317 PMC4585417

[41] Schneider CA, Rasband WS, Eliceiri KW. NIH Image to ImageJ: 25 years of image analysis. Nat Methods. 2012;9(7):671–5. 10.1038/nmeth.2089 22930834 PMC5554542

[42] Casadesus J, Villegas D. Conventional digital cameras as a tool for assessing leaf area index and biomass for cereal breeding. J Integr Plant Biol. 2014;56(1):7–14. 10.1111/jipb.12117 24330531

[43] Dreisigacker S, Tiwari R, Sheoran S. Laboratory manual: ICAR-CIMMYT molecular breeding course in wheat. In ICAR; 2013.

[44] Rasheed A, Wen W, Gao F, Zhai S, Jin H, Liu J, et al. Development and validation of KASP assays for genes underpinning key economic traits in bread wheat. Theor Appl Genet. 2016;129(10):1843–60. 10.1007/s00122-016-2743-x 27306516

[45] Giunta F, Motzo R, Deidda M. Effect of drought on yield and yield components of durum wheat and triticale in a Mediterranean environment. F Crop Res. 1993 6 1;33(4):399–409.

[46] Fischer RA, Maurer R. Drought resistance in spring wheat cultivars. I. Grain yield responses. Aust J Agric Res. 1978;29(5):897–912.

[47] Foulkes MJ, Sylvester-Bradley R, Weightman R, Snape JW. Identifying physiological traits associated with improved drought resistance in winter wheat. F Crop Res. 2007;103(1):11–24.

[48] Kumar BNA, Azam-Ali SN, Snape JW, Weightman RM, Foulkes MJ. Relationships between carbon isotope discrimination and grain yield in winter wheat under well-watered and drought conditions. J Agric Sci. 2011;149(3):257–72.

[49] Worland AJ, Börner A, Korzun V, Li WM, Petrovic S, Sayers EJ. The influence of photoperiod genes on the adaptability of European winter wheats. Euphytica. 1998;100(1–3):385–94.

[50] Wu X, Tang Y, Li C, Wu C. Characterization of the rate and duration of grain filling in wheat in southwestern China. Plant Prod Sci. 2018;21(4):358–69.

[51] Brdar MD, Kraljević-Balalić MM, Kobiljski BĐ. The parameters of grain filling and yield components in common wheat (Triticum aestivum L.) and durum wheat (Triticum turgidum L. var. durum). Cent Eur J Biol. 2008;3(1):75–82.

[52] Foulkes MJ, Sylvester-Bradley R, Worland AJ, Snape JW. Effects of a photoperiod-response gene Ppd-D1 on yield potential and drought resistance in UK winter wheat. Euphytica. 2004;135(1):63–73.

[53] Gorny AG, Garczynski S. Genotypic and nutrition-dependent variation in water use efficiency and photosynthetic activity of leaves in winter wheat (Triticum aestivum L.). J Appl Genet. 2002;43(2):145–60. 12080171

[54] Verma V, Foulkes MJ, Worland AJ, Sylvester-Bradley R, Caligari PDS, Snape JW. Mapping quantitative trait loci for flag leaf senescence as a yield determinant in winter wheat under optimal and drought-stressed environments. Euphytica. 2004;135(3):255–63.

[55] Christopher JT, Manschadi AM, Hammer GL, Borrell AK. Developmental and physiological traits associated with high yield and stay-green phenotype in wheat. Aust J Agric Res. 2008;59(4):354–64.

[56] Nehe AS, Misra S, Murchie EH, Chinnathambi K, Tyagi B, Foulkes MJ. Nitrogen partitioning and remobilization in relation to leaf senescence, grain yield and protein concentration in Indian wheat cultivars. F Crop Res. 2020 10.1016/j.fcr.2020.107778 32549650 PMC7182295

[57] Borrell AK, Hammer GL, Henzell RG. Does maintaining green leaf area in sorghum improve yield under drought? II. Dry matter production and yield. Crop Sci. 2000;40(4):1037–48.

[58] Nehe A, Akin B, Sanal T, Evlice AK, Ünsal R, Dinçer N, et al. Genotype x environment interaction and genetic gain for grain yield and grain quality traits in Turkish spring wheat released between 1964 and 2010. PLoS One. 2019 7 18;14(7):e0219432. 10.1371/journal.pone.0219432 31318895 PMC6638857

[59] Nehe AS, Misra S, Murchie EH, Chinnathambi K, Foulkes MJ. Genetic variation in N-use efficiency and associated traits in Indian wheat cultivars. F Crop Res [Internet]. 2018;225(June):152–62. Available from: 10.1016/j.fcr.2018.06.002 30078934 PMC6065306

[60] Burridge J, Jochua CN, Bucksch A, Lynch JP. Legume shovelomics: high—throughput phenotyping of common bean (Phaseolus vulgaris L.) and cowpea (Vigna unguiculata subsp, unguiculata) root architecture in the field. F Crop Res. 2016;192:21–32.

[61] El Hassouni K, Alahmad S, Belkadi B, Filali-Maltouf A, Hickey LT, Bassi FM. Root system architecture and its association with yield under different water regimes in durum wheat. Crop Sci. 2018;58(6):2331–46.

[62] Fan J, McConkey B, Wang H, Janzen H. Root distribution by depth for temperate agricultural crops. F Crop Res [Internet]. 2016;189:68–74. Available from: http://www.sciencedirect.com/science/article/pii/S0378429016300399

[63] Gao Y, Lynch JP. Reduced crown root number improves water acquisition under water deficit stress in maize (Zea mays L.). J Exp Bot. 2016;67(15):4545–57. 10.1093/jxb/erw243 27401910 PMC4973737

[64] Duggan BL, Richards RA, Van Herwaarden AF. Agronomic evaluation of a tiller inhibition gene (tin) in wheat. II. Growth and partitioning of assimilate. Aust J Agric Res. 2005;56(2):179–86.

[65] Richards RA. Physiological traits used in the breeding of new cultivars for water-scarce environments. Agric water Manag. 2006;80(1–3):197–211.

[66] Yoshida S, Hasegawa S. The rice root system: its development and function. Drought Resist Crop with Emphas rice. 1982;10:97–134.

[67] Wasaya A, Zhang X, Fang Q, Yan Z. Root phenotyping for drought tolerance: a review. Agronomy. 2018;8(11):241.

[68] Li P, Fan Y, Yin S, Wang Y, Wang H, Xu Y, et al. Multi-environment QTL mapping of crown root traits in a maize RIL population. Crop J. 2020

[69] Uga Y, Okuno K, Yano M. Dro1, a major QTL involved in deep rooting of rice under upland field conditions. J Exp Bot. 2011;62(8):2485–94. 10.1093/jxb/erq429 21212298

[70] Uga Y, Sugimoto K, Ogawa S, Rane J, Ishitani M, Hara N, et al. Control of root system architecture by DEEPER ROOTING 1 increases rice yield under drought conditions. Nat Genet. 2013;45(9):1097–102. 10.1038/ng.2725 23913002

[71] Grogan SM, Brown-Guedira G, Haley SD, McMaster GS, Reid SD, Smith J, et al. Allelic variation in developmental genes and effects on winter wheat heading date in the US Great Plains. PLoS One. 2016;11(4):e0152852. 10.1371/journal.pone.0152852 27058239 PMC4825937

[72] Perez-Lara E, Semagn K, Chen H, Ciechanowska I, Iqbal M, N’Diaye A, et al. Allelic variation and effects of 16 candidate genes on disease resistance in western Canadian spring wheat cultivars. Mol Breed. 2017;37(3):23.

[73] Zhang J, Dell B, Conocono E, Waters I, Setter T, Appels R. Water deficits in wheat: fructan exohydrolase (1‐FEH) mRNA expression and relationship to soluble carbohydrate concentrations in two varieties. New Phytol. 2009;181(4):843–50. 10.1111/j.1469-8137.2008.02713.x 19140945

[74] Shen Y-G, Zhang W-K, He S-J, Zhang J-S, Liu Q, Chen S-Y. An EREBP/AP2-type protein in Triticum aestivum was a DRE-binding transcription factor induced by cold, dehydration and ABA stress. Theor Appl Genet. 2003;106(5):923–30. 10.1007/s00122-002-1131-x 12647068

