# Supplementary material for: Root and canopy traits and adaptability genes explain drought tolerance responses in winter wheat
Source: PLoS One. 2021 Apr 5;16(4):e0242472. doi: 10.1371/journal.pone.0242472 (PMC8021186; doi:10.1371/journal.pone.0242472)
Supplement: S1 Table — (DOCX) [file pone.0242472.s001.docx]

**S1 Table.** Code number, genotype names, genotype origin country, grain yield (t ha^-1^) under IR and SA conditions, % yield reduction, and ranking of yield reduction for 50 advance breeding lines studied at Konya, Turkey in 2018 and 2019 under irrigated and semiarid conditions.

| Code | Genotype Name | Origin |
| --- | --- | --- |
| 1 | KONYA (irrigated-check) | TR-KON |
| 2 | NACIBEY (irrigated-semi-irrigated-check) | TCI-ESK |
| 3 | AFINA | RUS |
| 4 | LAUREAT | RUS |
| 5 | FISHT | RUS |
| 6 | DOLYA | RUS |
| 7 | DANPHE #1*2/SOLALA | MEX |
| 8 | REEDLING#1 | MEX |
| 9 | TAM-107//…/4/AGRI/NAC//ATTILA | TCI |
| 10 | KAPKA-I.P./MV10-2000//DORADE-5 | TCI |
| 11 | PFAU/MILAN//FUNG MAI 24/3/TAM200/KAUZ | TCI |
| 12 | ID800994.W/KAUZ// …/6/PANTHEON/BLUEGIL-2 | TCI |
| 13 | ALPU01/3/FDL4/KAUZ//LAGOS-7/4/…//CA8055 | TCI |
| 14 | CROC_1/AE.SQUARROSA …KS82142/PASTOR | TCI |
| 15 | KROSHKA/4/VORONA//MILAN/SHA7/3/MV17 | TCI |
| 16 | AiII192 | ROM |
| 17 | AiII198 | ROM |
| 18 | BiII98 | ROM |
| 19 | CH-211.13880 | CH |
| 20 | CH-211.14137 | CH |
| 21 | MOROZKO | RUS |
| 22 | KURS | RUS |
| 23 | UC1107(5+10;YR5;YR15;2NS)/SELYANKA | TCI |
| 24 | PATWIN YR15/4/VORONA/HD2402/3/…//CHAM6 | TCI |
| 25 | AXE//TOSUNBEY | TCI |
| 26 | PRL/2*PASTOR/3/KS82W409/SPN//…/TX78V3630 | TCI |
| 27 | DANPHE #1/6/CA8055/4/ …/5/AGRI/BJY//VEES | TCI |
| 28 | WHEAR//INQALAB 91*2/TUKURU/3/…//ATTILA | TCI |
| 29 | NIKIFOR//KROSHKA | TCI |
| 30 | NIKIFOR//KROSHKA | TCI |
| 31 | CBRD/TNMU//MILAN/3/…/4/IVETA NTA-92/89-6 | TCI |
| 32 | BABAX/LR42//BABAX*2/3/KURUKU/4/…/3/F10S-1 | TCI |
| 33 | BABAX/LR42//BABAX*2/3/…/AOS/3/F10S-1 | TCI |
| 34 | DORADE-5/3/PROGRESS//… /MOS83.11.4.8//PEW | TCI |
| 35 | D67.2/P66.270//AE.SQ./3/…/5/TX91D6991/B1551 | US-OK-TCI |
| 36 | TEMPORALERA*2/KONK/3/K92/…/TX96V2427 | US-OK-TCI |
| 37 | UC1110(5+10;YR15;YR15;GPC)/8/…/WA00477 | TCI |
| 38 | ID800994.W/KAUZ// …/6/PANTHEON/BLUEGIL-2 | TCI |
| 39 | VORONA/HD24-12//GUN/7/VEE#8//…/8/ALTAY | TCI |
| 40 | AGRI/NAC//ATTILA/6/…//TRAP#1/BOW | TCI |
| 41 | BILINMIYEN96.27//…/3/MNCH | TCI |
| 42 | BETTY/HEYNE//… /4/ATTILA//AGRI/NAC | MX-TCI |
| 43 | SHARORA//PBW343*2/KUKUNA | MX-TCI |
| 44 | ESPADA//KS82142/PASTOR | TCI |
| 45 | ALPU01/3/FDL4/KAUZ//…/3/HIM/CNDR//CA8055 | TCI |
| 46 | OBRII/DNESTREANCA25//ILICIOVCA/…/3/UN-49 | TCI |
| 47 | SAVALAN//…/4/2*F10S-1//STOZHER/KARL | TCI |
| 48 | ESPADA/KARAHAN | TCI |
| 49 | GEREK (semi-irrigated-check) | TR-ESK |
| 50 | KATEA (irrigated-check) | TR-EDR |

G: Genotype, T: Treatment, Y: Years, TR-KON: Turkey-Konya, TCI-ESK: Turkey CIMMYT ICARDA- Eskisehir, RUS: Russia, MEX: Mexico, TCI: Turkey CIMMYT ICARDA, ROM: Romania, CH: China, MX-TCI: Mexico-Turkey CIMMYT-ICARDA, TR-ESK: Turkey- Eskisehir.
