# Supplementary material for: Root and canopy traits and adaptability genes explain drought tolerance responses in winter wheat
Source: PLoS One. 2021 Apr 5;16(4):e0242472. doi: 10.1371/journal.pone.0242472 (PMC8021186; doi:10.1371/journal.pone.0242472)
Supplement: S2 Table — (DOCX) [file pone.0242472.s002.docx]

**S2 Table.** Mean, maximum, minimum, LSD for grain yield (GY), percent GY reduction under SA (%Red), above ground dry matter (AGDM), thousand grain weight (TKW), harvest index (HI), plant height (PH), heading date (HD), ear length (EarL), spikelets per ear (Spkt Ear^-1^), fruiting efficiency at harvest (FE), NDVI at anthesis (NDVI) for 50 genotypes under IR and SA conditions(mean of 2018 and 2019).

|  | GY t ha^-1^ | | %Red | AGDM t ha^-1^ | | TKW g | | HI | | PH cm | | HD DAS | | EarL cm | | SpktNo Ear^-1^ | | FE grains/g | | NDVI | |
| --- | --- | --- | --- | --- | --- | --- | --- | --- | --- | --- | --- | --- | --- | --- | --- | --- | --- | --- | --- | --- | --- |
| ID | IR | SA |  | IR | SA | IR | SA | IR | SA | IR | SA | IR | SA | IR | SA | IR | SA | IR | SA | IR | SA |
| 1 | 5.46 | 3.69 | 32.4 | 18.5 | 11.5 | 40.4 | 37.3 | 0.35 | 0.35 | 88.4 | 58.1 | 181 | 181 | 10.6 | 10.6 | 18.8 | 19.4 | 15.0 | 17.0 | 0.61 | 0.53 |
| 2 | 6.96 | 3.80 | 45.4 | 19.4 | 13.2 | 38.0 | 33.5 | 0.38 | 0.35 | 91.0 | 66.1 | 182 | 181 | 9.7 | 8.7 | 17.6 | 17.3 | 17.3 | 17.6 | 0.65 | 0.55 |
| 3 | 7.32 | 3.48 | 52.5 | 21.4 | 13.1 | 32.6 | 30.3 | 0.37 | 0.32 | 83.3 | 59.7 | 182 | 181 | 10.2 | 10.3 | 19.2 | 19.9 | 20.2 | 19.0 | 0.69 | 0.57 |
| 4 | 6.79 | 3.87 | 43.0 | 22.2 | 11.6 | 33.6 | 28.7 | 0.36 | 0.39 | 78.7 | 53.1 | 183 | 181 | 10.6 | 10.1 | 18.8 | 19.5 | 17.3 | 21.4 | 0.71 | 0.61 |
| 5 | 6.92 | 3.87 | 44.1 | 18.8 | 11.3 | 32.3 | 30.1 | 0.40 | 0.40 | 72.1 | 53.1 | 181 | 180 | 8.6 | 9.0 | 19.5 | 20.6 | 20.8 | 22.2 | 0.69 | 0.61 |
| 6 | 6.67 | 2.77 | 58.5 | 17.5 | 9.2 | 37.4 | 33.3 | 0.39 | 0.32 | 86.1 | 56.2 | 187 | 184 | 10.8 | 8.8 | 19.9 | 17.7 | 17.9 | 18.0 | 0.69 | 0.57 |
| 7 | 7.44 | 3.97 | 46.7 | 19.3 | 11.0 | 44.7 | 36.5 | 0.40 | 0.39 | 90.1 | 62.8 | 180 | 178 | 9.6 | 9.7 | 18.8 | 19.3 | 15.1 | 17.8 | 0.66 | 0.55 |
| 8 | 6.86 | 3.67 | 46.5 | 19.3 | 11.2 | 39.5 | 35.5 | 0.34 | 0.37 | 81.1 | 52.2 | 176 | 176 | 9.4 | 10.0 | 17.3 | 18.2 | 14.8 | 17.6 | 0.63 | 0.53 |
| 9 | 6.58 | 2.78 | 57.8 | 21.7 | 14.6 | 34.7 | 31.3 | 0.33 | 0.23 | 88.2 | 55.8 | 180 | 178 | 8.8 | 8.8 | 17.8 | 18.0 | 16.5 | 16.0 | 0.69 | 0.50 |
| 10 | 7.08 | 3.39 | 52.1 | 19.3 | 10.6 | 37.4 | 34.9 | 0.39 | 0.37 | 82.7 | 57.1 | 182 | 180 | 10.5 | 10.5 | 20.1 | 20.9 | 17.7 | 17.5 | 0.70 | 0.58 |
| 11 | 7.41 | 3.70 | 50.0 | 19.3 | 12.5 | 37.9 | 32.2 | 0.41 | 0.36 | 87.7 | 60.0 | 185 | 181 | 10.5 | 10.4 | 15.5 | 15.8 | 17.9 | 18.7 | 0.74 | 0.58 |
| 12 | 5.81 | 3.11 | 46.4 | 17.2 | 11.7 | 36.8 | 32.5 | 0.35 | 0.31 | 84.6 | 63.3 | 184 | 181 | 11.2 | 10.6 | 19.1 | 18.8 | 16.9 | 17.0 | 0.66 | 0.54 |
| 13 | 6.40 | 3.26 | 49.1 | 18.0 | 11.1 | 39.5 | 31.2 | 0.37 | 0.35 | 85.5 | 54.1 | 180 | 180 | 11.7 | 10.6 | 18.8 | 19.1 | 16.3 | 19.0 | 0.65 | 0.54 |
| 14 | 6.76 | 3.45 | 49.0 | 19.6 | 12.2 | 35.1 | 27.3 | 0.39 | 0.34 | 84.7 | 60.7 | 179 | 177 | 10.6 | 9.7 | 18.6 | 18.4 | 18.6 | 22.1 | 0.68 | 0.53 |
| 15 | 6.18 | 3.19 | 48.4 | 19.3 | 11.0 | 39.4 | 33.0 | 0.36 | 0.35 | 77.1 | 48.2 | 183 | 181 | 8.8 | 9.0 | 17.6 | 18.0 | 15.9 | 18.1 | 0.62 | 0.50 |
| 16 | 6.42 | 4.18 | 34.8 | 20.1 | 12.0 | 42.5 | 37.0 | 0.36 | 0.39 | 77.1 | 52.3 | 179 | 178 | 8.3 | 9.0 | 15.7 | 15.8 | 15.0 | 17.6 | 0.65 | 0.59 |
| 17 | 6.86 | 3.93 | 42.7 | 20.4 | 12.0 | 39.1 | 34.6 | 0.35 | 0.38 | 72.5 | 48.7 | 179 | 178 | 9.0 | 9.5 | 14.9 | 16.3 | 15.7 | 18.0 | 0.66 | 0.55 |
| 18 | 7.11 | 4.29 | 39.6 | 23.6 | 14.5 | 38.3 | 32.7 | 0.35 | 0.34 | 83.8 | 55.0 | 179 | 178 | 8.6 | 8.9 | 16.7 | 17.7 | 15.8 | 18.6 | 0.68 | 0.55 |
| 19 | 6.62 | 2.97 | 55.1 | 23.2 | 15.3 | 31.1 | 26.4 | 0.30 | 0.24 | 90.0 | 64.8 | 185 | 181 | 10.4 | 9.4 | 21.7 | 20.9 | 19.2 | 19.1 | 0.66 | 0.58 |
| 20 | 5.59 | 2.91 | 48.0 | 20.7 | 11.8 | 26.6 | 25.7 | 0.28 | 0.29 | 74.5 | 53.3 | 188 | 186 | 9.6 | 9.2 | 19.5 | 19.8 | 21.4 | 21.1 | 0.69 | 0.56 |
| 21 | 6.04 | 3.17 | 47.5 | 17.6 | 10.3 | 36.2 | 31.7 | 0.38 | 0.37 | 83.2 | 52.7 | 184 | 181 | 10.2 | 9.9 | 19.5 | 19.4 | 18.0 | 20.1 | 0.66 | 0.52 |
| 22 | 6.08 | 3.12 | 48.7 | 16.8 | 9.8 | 38.7 | 32.7 | 0.39 | 0.36 | 79.8 | 55.7 | 186 | 181 | 9.2 | 9.1 | 19.4 | 19.4 | 18.1 | 19.6 | 0.67 | 0.54 |
| 23 | 7.02 | 4.07 | 42.0 | 21.3 | 14.4 | 31.9 | 31.3 | 0.37 | 0.34 | 80.1 | 58.2 | 180 | 179 | 9.9 | 10.1 | 18.3 | 19.2 | 19.3 | 18.2 | 0.68 | 0.56 |
| 24 | 6.88 | 3.19 | 53.7 | 19.0 | 14.2 | 34.3 | 28.0 | 0.38 | 0.29 | 78.2 | 52.2 | 183 | 183 | 9.3 | 9.3 | 18.9 | 20.0 | 19.3 | 18.5 | 0.67 | 0.48 |
| 25 | 6.98 | 4.17 | 40.2 | 18.7 | 15.9 | 33.8 | 30.9 | 0.39 | 0.31 | 81.5 | 63.0 | 178 | 177 | 8.7 | 9.0 | 17.2 | 17.2 | 20.3 | 18.7 | 0.66 | 0.52 |
| 26 | 7.24 | 3.40 | 53.1 | 19.1 | 11.0 | 45.7 | 34.0 | 0.40 | 0.34 | 87.7 | 58.4 | 181 | 178 | 9.7 | 9.7 | 15.8 | 16.5 | 14.5 | 17.1 | 0.72 | 0.53 |
| 27 | 6.02 | 3.78 | 37.2 | 18.3 | 12.4 | 38.4 | 33.9 | 0.35 | 0.35 | 86.5 | 63.2 | 180 | 179 | 8.9 | 9.0 | 17.6 | 18.1 | 15.9 | 17.8 | 0.63 | 0.58 |
| 28 | 7.20 | 3.62 | 49.8 | 19.4 | 13.7 | 37.5 | 34.3 | 0.39 | 0.34 | 90.3 | 60.4 | 180 | 180 | 9.5 | 9.7 | 19.6 | 20.5 | 19.1 | 16.4 | 0.67 | 0.55 |
| 29 | 7.81 | 4.19 | 46.4 | 20.4 | 12.7 | 38.6 | 33.9 | 0.41 | 0.37 | 85.3 | 58.2 | 181 | 180 | 10.6 | 10.3 | 17.6 | 18.0 | 17.9 | 18.9 | 0.65 | 0.58 |
| 30 | 6.58 | 3.36 | 49.0 | 20.4 | 10.4 | 41.4 | 32.6 | 0.36 | 0.39 | 83.4 | 60.9 | 181 | 178 | 11.8 | 12.0 | 17.4 | 17.5 | 15.5 | 20.0 | 0.67 | 0.56 |
| 31 | 6.86 | 3.98 | 42.0 | 20.8 | 13.9 | 38.1 | 37.7 | 0.35 | 0.31 | 89.9 | 66.5 | 183 | 182 | 10.9 | 11.6 | 20.3 | 21.0 | 16.5 | 15.4 | 0.70 | 0.60 |
| 32 | 5.86 | 3.74 | 36.1 | 17.3 | 14.0 | 36.4 | 33.5 | 0.36 | 0.31 | 78.6 | 57.9 | 182 | 181 | 10.3 | 9.9 | 19.0 | 19.2 | 17.4 | 16.7 | 0.65 | 0.56 |
| 33 | 7.73 | 3.92 | 49.3 | 23.8 | 12.1 | 32.3 | 30.0 | 0.35 | 0.35 | 81.8 | 58.2 | 182 | 179 | 9.8 | 9.8 | 19.5 | 19.8 | 18.6 | 21.0 | 0.71 | 0.53 |
| 34 | 5.55 | 3.03 | 45.4 | 19.5 | 10.0 | 34.3 | 33.6 | 0.32 | 0.33 | 75.5 | 46.8 | 185 | 181 | 10.4 | 9.7 | 19.0 | 18.7 | 16.1 | 16.7 | 0.72 | 0.52 |
| 35 | 5.25 | 2.64 | 49.8 | 17.7 | 9.6 | 32.8 | 29.0 | 0.32 | 0.32 | 88.4 | 56.4 | 183 | 180 | 10.0 | 9.4 | 17.3 | 17.9 | 18.2 | 18.8 | 0.66 | 0.53 |
| 36 | 6.13 | 3.45 | 43.7 | 18.1 | 13.1 | 37.9 | 33.5 | 0.36 | 0.31 | 84.0 | 59.4 | 185 | 183 | 10.5 | 10.2 | 17.8 | 18.1 | 16.6 | 16.8 | 0.71 | 0.59 |
| 37 | 6.64 | 3.88 | 41.5 | 17.9 | 11.7 | 32.6 | 30.9 | 0.39 | 0.36 | 81.0 | 49.6 | 182 | 179 | 11.2 | 10.7 | 20.0 | 14.5 | 20.1 | 19.8 | 0.70 | 0.52 |
| 38 | 6.47 | 2.96 | 54.2 | 19.0 | 9.6 | 37.7 | 36.3 | 0.37 | 0.33 | 87.5 | 61.2 | 182 | 180 | 10.3 | 10.2 | 17.5 | 18.7 | 17.1 | 17.1 | 0.65 | 0.52 |
| 39 | 7.39 | 4.37 | 40.9 | 21.9 | 13.1 | 40.9 | 36.0 | 0.35 | 0.37 | 93.6 | 68.2 | 182 | 180 | 10.0 | 9.3 | 17.4 | 17.7 | 14.7 | 17.7 | 0.65 | 0.56 |
| 40 | 5.83 | 2.48 | 57.5 | 18.6 | 10.7 | 34.1 | 33.4 | 0.34 | 0.27 | 83.9 | 58.4 | 183 | 180 | 11.4 | 9.7 | 19.5 | 17.2 | 17.8 | 16.0 | 0.67 | 0.45 |
| 41 | 6.28 | 3.65 | 41.9 | 23.1 | 10.7 | 32.1 | 29.3 | 0.32 | 0.38 | 75.8 | 53.1 | 181 | 180 | 8.0 | 9.3 | 17.7 | 19.0 | 17.1 | 21.3 | 0.65 | 0.52 |
| 42 | 6.06 | 2.95 | 51.4 | 19.5 | 12.4 | 40.3 | 34.5 | 0.34 | 0.29 | 84.0 | 52.2 | 183 | 181 | 10.2 | 9.4 | 19.1 | 18.1 | 15.1 | 14.8 | 0.64 | 0.49 |
| 43 | 6.73 | 2.94 | 56.3 | 20.3 | 10.9 | 29.4 | 27.2 | 0.36 | 0.33 | 85.1 | 57.1 | 184 | 181 | 10.9 | 10.2 | 21.4 | 21.0 | 20.7 | 20.2 | 0.68 | 0.53 |
| 44 | 6.32 | 3.37 | 46.7 | 18.8 | 12.5 | 33.1 | 28.6 | 0.36 | 0.31 | 85.8 | 55.2 | 179 | 179 | 8.8 | 8.6 | 18.8 | 19.7 | 19.3 | 20.6 | 0.64 | 0.47 |
| 45 | 6.76 | 2.94 | 56.5 | 19.8 | 10.9 | 39.4 | 30.1 | 0.38 | 0.34 | 88.8 | 53.2 | 181 | 181 | 12.1 | 11.4 | 18.9 | 19.0 | 16.3 | 18.7 | 0.67 | 0.52 |
| 46 | 6.91 | 3.55 | 48.6 | 18.5 | 11.4 | 33.6 | 27.8 | 0.38 | 0.36 | 80.3 | 50.8 | 184 | 181 | 9.4 | 8.9 | 18.2 | 17.8 | 20.2 | 22.1 | 0.68 | 0.52 |
| 47 | 7.44 | 4.03 | 45.8 | 21.8 | 12.7 | 42.4 | 34.1 | 0.39 | 0.36 | 89.6 | 62.6 | 182 | 180 | 9.9 | 9.5 | 17.7 | 17.9 | 15.2 | 17.9 | 0.72 | 0.58 |
| 48 | 7.05 | 4.14 | 41.3 | 20.3 | 13.1 | 38.9 | 33.8 | 0.36 | 0.34 | 79.1 | 52.8 | 184 | 180 | 9.7 | 9.6 | 17.6 | 18.6 | 15.8 | 17.8 | 0.71 | 0.53 |
| 49 | 5.43 | 3.96 | 27.0 | 19.5 | 15.2 | 32.6 | 29.0 | 0.29 | 0.31 | 94.6 | 65.3 | 181 | 180 | 8.0 | 8.1 | 15.8 | 16.3 | 17.1 | 19.6 | 0.72 | 0.56 |
| 50 | 6.78 | 3.49 | 48.5 | 21.6 | 12.2 | 35.4 | 28.8 | 0.35 | 0.35 | 84.0 | 60.5 | 180 | 179 | 9.7 | 10.0 | 18.1 | 18.7 | 17.6 | 20.9 | 0.65 | 0.54 |
|  |  |  |  |  |  |  |  |  |  |  |  |  |  |  |  |  |  |  |  |  |  |
| Mean | 6.61 | 3.51 |  | 83.7 | 57.3 | 36.6 | 32.1 | 0.36 | 0.34 | 83.7 | 57.3 | 182 | 180 | 9.9 | 9.7 | 18.3 | 18.5 | 17.4 | 18.6 | 0.67 | 0.54 |
| Min | 5.25 | 2.48 |  | 72.1 | 46.8 | 26.6 | 25.7 | 0.28 | 0.23 | 72.1 | 46.8 | 176 | 176 | 8.0 | 8.1 | 14.9 | 14.5 | 14.5 | 14.8 | 0.61 | 0.45 |
| Max | 7.81 | 4.37 |  | 94.6 | 68.2 | 45.7 | 37.7 | 0.41 | 0.40 | 94.6 | 68.2 | 188 | 186 | 12.1 | 12.0 | 21.7 | 21.0 | 21.4 | 22.2 | 0.74 | 0.61 |
|  | LSD |  |  | LSD |  | LSD |  | LSD |  | LSD |  | LSD |  | LSD |  | LSD |  | LSD |  | LSD |  |
| G | 0.70 | *** |  | 2.95 | ** | 2.81 | *** | 0.04 | *** | 4.21 | *** | 1.40 | *** | 0.57 | *** | 1.11 | *** | 1.86 | *** | 0.04 | *** |
| T | 1.47 | ** |  | 2.90 | ** | 3.84 | * | 0.01 | * | 7.56 | ** | 0.50 | ** | 0.64 |  | 0.75 |  | 1.80 |  | 0.03 | ** |
| Y | 0.60 | ** |  | 2.24 | ** | 1.27 | * | 0.05 | ** | 5.48 | * | 1.50 | *** | 0.33 | ** | 0.82 | ** | 1.25 | ** | 0.03 | ** |
| T*G | 1.27 |  |  | 4.35 |  | 4.38 | * | 0.06 |  | 7.10 | * | 1.90 | ** | 0.85 | *** | 1.59 | *** | 2.74 | ** | 0.06 | ** |
| Y*G | 1.02 | *** |  | 4.25 |  | 3.98 | *** | 0.06 | * | 6.49 |  | 2.10 | *** | 0.81 | ** | 1.60 | ** | 2.67 | *** | 0.06 | ** |

Significance levels displayed as * <.05 >.01, ** <.01, ***<0.001.
