# Supplementary material for: Root and canopy traits and adaptability genes explain drought tolerance responses in winter wheat
Source: PLoS One. 2021 Apr 5;16(4):e0242472. doi: 10.1371/journal.pone.0242472 (PMC8021186; doi:10.1371/journal.pone.0242472)
Supplement: S3 Table — (DOCX) [file pone.0242472.s003.docx]

**S3 Table.** Traits Definition and a list of traits measured in 2019, which section of a crown measured from, and their derivations.

| Traits | Definition |
| --- | --- |
| Shoot number | Average shoot number per plant counted from 3 plants |
| Root number | Average of total visible roots per plant in 2D image from 3 plants |
| Stem width | Average width at base of all stems measured |
| System width | Width of the root system taken horizontally in the middle of root system |
| Depth-to-width | The vertical distance from center of crown root to system width |
| Root angle | Derived from the system width, and depth-to-width |
| Root diameter | Width average overall 10 measurements per plant taken at widest point of representative root |
