# Supplementary material for: Root and canopy traits and adaptability genes explain drought tolerance responses in winter wheat
Source: PLoS One. 2021 Apr 5;16(4):e0242472. doi: 10.1371/journal.pone.0242472 (PMC8021186; doi:10.1371/journal.pone.0242472)
Supplement: S4 Table — (DOCX) [file pone.0242472.s004.docx]

**S4 Table.** Correlation matrix showing correlation coefficient (r) values and *P*-values (in orange color) for grain yield (GY), above ground dry matter (AGDM), harvest index (HI), thousand grain weight (TGW), heading date (HD), root surface area (RoSuAr), root diameter (RoDiM), root volume (RoVol), NDVI at anthesis (NDVI), NDVI senescence start (SenSt), NDVI senescence duration (SenDu) for selected 30 genotypes with one day difference in flowering date under irrigated (IR) and semiarid (SA) conditions for 2018.

|  | **GY** |  | **AGDM** |  | **HI** |  | **TKW** |  | **HD** |  | **RoSuAr** |  | **RoDiM** |  | **RoVol** |  | **NDVI** |  | **SenSt** |  |
| --- | --- | --- | --- | --- | --- | --- | --- | --- | --- | --- | --- | --- | --- | --- | --- | --- | --- | --- | --- | --- |
|  |  |  |  |  |  |  |  |  |  |  |  |  |  |  |  |  |  |  |  |  |
| **IR** |  |  |  |  |  |  |  |  |  |  |  |  |  |  |  |  |  |  |  |  |
| GY | - | - |  |  |  |  |  |  |  |  |  |  |  |  |  |  |  |  |  |  |
| AGDM | 0.80 | <0.001 | - | - |  |  |  |  |  |  |  |  |  |  |  |  |  |  |  |  |
| HI | 0.09 | 0.630 | -0.51 | 0.00 | - | - |  |  |  |  |  |  |  |  |  |  |  |  |  |  |
| TKW | 0.15 | 0.430 | 0.04 | 0.84 | 0.17 | 0.37 | - | - |  |  |  |  |  |  |  |  |  |  |  |  |
| PH | 0.22 | 0.251 | 0.35 | 0.06 | -0.29 | 0.12 | 0.50 | 0.01 |  |  |  |  |  |  |  |  |  |  |  |  |
| RoSuAr | 0.05 | 0.797 | -0.12 | 0.52 | 0.30 | 0.11 | 0.28 | 0.14 | 0.22 | 0.25 | - | - |  |  |  |  |  |  |  |  |
| RoDiM | -0.03 | 0.870 | -0.08 | 0.67 | 0.00 | 0.99 | 0.04 | 0.83 | -0.44 | 0.02 | 0.06 | 0.77 | - | - |  |  |  |  |  |  |
| RoVol | 0.04 | 0.834 | -0.11 | 0.56 | 0.23 | 0.24 | 0.28 | 0.15 | -0.03 | 0.87 | 0.86 | <0.001 | 0.55 | 0.00 | - | - |  |  |  |  |
| NDVI | 0.45 | 0.015 | 0.46 | 0.01 | -0.11 | 0.57 | -0.15 | 0.45 | 0.32 | 0.09 | -0.20 | 0.30 | -0.26 | 0.17 | -0.28 | 0.14 | - | - |  |  |
| SenSt | 0.24 | 0.211 | 0.21 | 0.27 | -0.03 | 0.87 | 0.27 | 0.15 | -0.25 | 0.20 | 0.05 | 0.79 | 0.19 | 0.32 | 0.13 | 0.50 | -0.20 | 0.29 | - | - |
| SenDu | -0.07 | 0.700 | 0.00 | 0.98 | -0.09 | 0.65 | -0.20 | 0.29 | -0.15 | 0.43 | -0.25 | 0.20 | -0.04 | 0.83 | -0.21 | 0.27 | 0.13 | 0.51 | -0.75 | <0.001 |
|  |  |  |  |  |  |  |  |  |  |  |  |  |  |  |  |  |  |  |  |  |
|  |  |  |  |  |  |  |  |  |  |  |  |  |  |  |  |  |  |  |  |  |
|  |  |  |  |  |  |  |  |  |  |  |  |  |  |  |  |  |  |  |  |  |
| **SA** |  |  |  |  |  |  |  |  |  |  |  |  |  |  |  |  |  |  |  |  |
| GY | - | - |  |  |  |  |  |  |  |  |  |  |  |  |  |  |  |  |  |  |
| AGDM | 0.91 | <0.001 | - | - |  |  |  |  |  |  |  |  |  |  |  |  |  |  |  |  |
| HI | 0.45 | 0.015 | 0.05 | 0.79 | - | - |  |  |  |  |  |  |  |  |  |  |  |  |  |  |
| TKW | -0.09 | 0.655 | -0.06 | 0.75 | -0.10 | 0.61 | - | - |  |  |  |  |  |  |  |  |  |  |  |  |
| PH | 0.53 | 0.003 | 0.60 | <0.001 | 0.01 | 0.96 | 0.46 | 0.01 |  |  |  |  |  |  |  |  |  |  |  |  |
| RoSuAr | -0.34 | 0.069 | -0.38 | 0.04 | -0.04 | 0.85 | 0.40 | 0.03 | 0.18 | 0.34 | - | - |  |  |  |  |  |  |  |  |
| RoDiM | -0.35 | 0.066 | -0.35 | 0.07 | -0.08 | 0.68 | 0.45 | 0.02 | 0.13 | 0.51 | 0.48 | 0.01 | - | - |  |  |  |  |  |  |
| RoVol | -0.37 | 0.051 | -0.38 | 0.04 | -0.08 | 0.69 | 0.42 | 0.02 | 0.22 | 0.26 | 0.93 | <0.001 | 0.73 | <0.001 | - | - |  |  |  |  |
| NDVI | 0.55 | 0.002 | 0.28 | 0.14 | 0.73 | <0.001 | -0.13 | 0.49 | -0.08 | 0.66 | -0.05 | 0.81 | -0.26 | 0.17 | -0.12 | 0.52 | - | - |  |  |
| SenSt | -0.09 | 0.661 | -0.33 | 0.08 | 0.56 | 0.00 | -0.07 | 0.74 | 0.14 | 0.47 | -0.01 | 0.94 | -0.01 | 0.94 | 0.01 | 0.96 | 0.52 | 0.00 | - | - |
| SenDu | -0.07 | 0.710 | 0.15 | 0.43 | -0.56 | 0.00 | 0.03 | 0.86 | -0.22 | 0.26 | 0.06 | 0.75 | -0.03 | 0.87 | 0.01 | 0.94 | -0.57 | 0.00 | -0.78 | <0.001 |
