# Supplementary material for: Root and canopy traits and adaptability genes explain drought tolerance responses in winter wheat
Source: PLoS One. 2021 Apr 5;16(4):e0242472. doi: 10.1371/journal.pone.0242472 (PMC8021186; doi:10.1371/journal.pone.0242472)
Supplement: S5 Table — (DOCX) [file pone.0242472.s005.docx]

**S5 Table.** Correlation matrix showing correlation coefficient (r) values and *P*-values (in orange color) for grain yield (GY), above ground dry matter (AGDM), harvest index (HI), thousand grain weight (TGW), days to heading (DH), canopy green area per meter square at anthesis (GA An) and after 2 weeks of anthesis (GA 2W), NDVI at anthesis (NDVI), root angle (RoAng), root diameter (RoDiM), root dry weight per plant (RoDrWtPl), and canopy temperature at anthesis (CT) for selected 30 genotypes with one day difference in flowering date under irrigated (IR) and semiarid (SA) conditions for 2019.

|  | **GY** |  | **AGDM** |  | **HI** |  | **TGW** |  | **HD** |  | **GA An** |  | **GA 2W** |  | **NDVI** |  | **RoAng** |  | **RoDiM** |  | **RoDrWtPl** |  | **RoNoPl** |  |
| --- | --- | --- | --- | --- | --- | --- | --- | --- | --- | --- | --- | --- | --- | --- | --- | --- | --- | --- | --- | --- | --- | --- | --- | --- |
|  |  |  |  |  |  |  |  |  |  |  |  |  |  |  |  |  |  |  |  |  |  |  |  |  |
| **IR** |  |  |  |  |  |  |  |  |  |  |  |  |  |  |  |  |  |  |  |  |  |  |  |  |
| GY | - | - |  |  |  |  |  |  |  |  |  |  |  |  |  |  |  |  |  |  |  |  |  |  |
| AGDM | 0.48 | 0.01 | - | - |  |  |  |  |  |  |  |  |  |  |  |  |  |  |  |  |  |  |  |  |
| HI | 0.48 | 0.02 | -0.52 | 0.01 | - | - |  |  |  |  |  |  |  |  |  |  |  |  |  |  |  |  |  |  |
| TGW | 0.06 | 0.76 | -0.26 | 0.21 | 0.23 | 0.26 | - | - |  |  |  |  |  |  |  |  |  |  |  |  |  |  |  |  |
| HD | -0.13 | 0.54 | -0.20 | 0.35 | 0.07 | 0.74 | 0.32 | 0.12 | - | - |  |  |  |  |  |  |  |  |  |  |  |  |  |  |
| GA An | 0.51 | 0.01 | 0.18 | 0.40 | 0.39 | 0.05 | -0.20 | 0.34 | 0.07 | 0.74 | - | - |  |  |  |  |  |  |  |  |  |  |  |  |
| GA 2W | 0.46 | 0.02 | 0.15 | 0.46 | 0.33 | 0.11 | -0.12 | 0.58 | 0.15 | 0.46 | 0.89 | <0.001 | - | - |  |  |  |  |  |  |  |  |  |  |
| NDVI | 0.41 | 0.04 | 0.15 | 0.49 | 0.33 | 0.11 | -0.19 | 0.36 | 0.35 | 0.09 | 0.67 | <0.001 | 0.70 | <0.001 | - | - |  |  |  |  |  |  |  |  |
| RoAng | 0.26 | 0.20 | 0.17 | 0.43 | 0.05 | 0.80 | -0.04 | 0.85 | 0.04 | 0.86 | 0.01 | 0.97 | 0.08 | 0.71 | 0.38 | 0.06 | - | - |  |  |  |  |  |  |
| RoDiM | -0.13 | 0.53 | -0.42 | 0.03 | 0.30 | 0.15 | 0.00 | 0.99 | 0.43 | 0.03 | 0.12 | 0.58 | 0.19 | 0.37 | 0.43 | 0.03 | 0.14 | 0.51 | - | - |  |  |  |  |
| RoDrWtPl | -0.18 | 0.39 | -0.34 | 0.09 | 0.23 | 0.26 | 0.12 | 0.55 | 0.38 | 0.06 | 0.29 | 0.16 | 0.34 | 0.09 | 0.30 | 0.14 | -0.20 | 0.34 | 0.38 | 0.06 | - | - |  |  |
| RoNoPl | -0.17 | 0.43 | 0.22 | 0.28 | -0.34 | 0.09 | -0.14 | 0.51 | 0.31 | 0.13 | 0.02 | 0.91 | 0.03 | 0.87 | 0.24 | 0.26 | -0.08 | 0.71 | 0.28 | 0.18 | 0.39 | 0.06 | - | - |
| CT | 0.12 | 0.57 | -0.21 | 0.32 | 0.20 | 0.33 | 0.15 | 0.47 | 0.14 | 0.51 | -0.03 | 0.89 | -0.08 | 0.70 | -0.12 | 0.56 | -0.02 | 0.93 | 0.20 | 0.35 | -0.06 | 0.79 | -0.04 | 0.87 |
|  |  |  |  |  |  |  |  |  |  |  |  |  |  |  |  |  |  |  |  |  |  |  |  |  |
|  |  |  |  |  |  |  |  |  |  |  |  |  |  |  |  |  |  |  |  |  |  |  |  |  |
| **SA** |  |  |  |  |  |  |  |  |  |  |  |  |  |  |  |  |  |  |  |  |  |  |  |  |
| GY | - | - |  |  |  |  |  |  |  |  |  |  |  |  |  |  |  |  |  |  |  |  |  |  |
| AGDM | 0.58 | 0.00 | - | - |  |  |  |  |  |  |  |  |  |  |  |  |  |  |  |  |  |  |  |  |
| HI | 0.62 | 0.00 | -0.27 | 0.18 | - | - |  |  |  |  |  |  |  |  |  |  |  |  |  |  |  |  |  |  |
| TGW | 0.45 | 0.02 | 0.10 | 0.64 | 0.45 | 0.02 | - | - |  |  |  |  |  |  |  |  |  |  |  |  |  |  |  |  |
| HD | -0.40 | 0.05 | -0.25 | 0.23 | -0.24 | 0.24 | 0.18 | 0.38 | - | - |  |  |  |  |  |  |  |  |  |  |  |  |  |  |
| GA An | 0.62 | 0.00 | 0.37 | 0.07 | 0.35 | 0.09 | 0.32 | 0.12 | -0.14 | 0.50 | - | - |  |  |  |  |  |  |  |  |  |  |  |  |
| GA 2W | 0.82 | <0.001 | 0.35 | 0.08 | 0.61 | 0.00 | 0.46 | 0.02 | -0.18 | 0.40 | 0.76 | <0.001 | - | - |  |  |  |  |  |  |  |  |  |  |
| NDVI | 0.56 | 0.00 | 0.16 | 0.44 | 0.47 | 0.02 | 0.36 | 0.07 | -0.11 | 0.60 | 0.90 | <0.001 | 0.76 | <0.001 | - | - |  |  |  |  |  |  |  |  |
| RoAng | 0.25 | 0.22 | 0.10 | 0.64 | 0.13 | 0.53 | -0.04 | 0.84 | 0.05 | 0.82 | -0.02 | 0.91 | 0.16 | 0.44 | -0.01 | 0.97 | - | - |  |  |  |  |  |  |
| RoDiM | -0.27 | 0.19 | -0.04 | 0.85 | -0.28 | 0.18 | -0.18 | 0.39 | -0.22 | 0.29 | -0.29 | 0.17 | -0.42 | 0.04 | -0.14 | 0.51 | 0.05 | 0.83 | - | - |  |  |  |  |
| RoDrWtPl | -0.29 | 0.15 | -0.13 | 0.52 | -0.18 | 0.39 | -0.10 | 0.65 | 0.14 | 0.52 | -0.37 | 0.07 | -0.33 | 0.11 | -0.24 | 0.24 | -0.44 | 0.03 | -0.02 | 0.92 | - | - |  |  |
| RoNoPl | -0.06 | 0.78 | -0.10 | 0.63 | 0.03 | 0.88 | 0.05 | 0.81 | 0.26 | 0.21 | -0.30 | 0.15 | -0.12 | 0.58 | -0.20 | 0.33 | -0.32 | 0.12 | -0.17 | 0.42 | 0.74 | <0.001 | - | - |
| CT | -0.37 | 0.07 | -0.33 | 0.11 | -0.12 | 0.57 | -0.16 | 0.45 | 0.02 | 0.92 | -0.38 | 0.06 | -0.42 | 0.04 | -0.30 | 0.15 | -0.25 | 0.22 | 0.22 | 0.29 | 0.11 | 0.59 | -0.07 | 0.73 |
