# Supplementary material for: Root and canopy traits and adaptability genes explain drought tolerance responses in winter wheat
Source: PLoS One. 2021 Apr 5;16(4):e0242472. doi: 10.1371/journal.pone.0242472 (PMC8021186; doi:10.1371/journal.pone.0242472)
Supplement: S6 Table — Stepwise multi-linear regression with grain yield (GY) as the dependent variable showing the best model for selected traits in irrigated (IR) and semiarid (SA) conditions in 2018 and 2019: Grain yield (GY), root surface area (RoSuAr), root diameter (RoDiM), root volume (RoVol), NDVI at anthesis (NDVI), NDVI senescence start (SenSt), NDVI senescence duration (SenDu) in 2018 and root angle (RoAng), root diameter (RoDiM), root dry weight per shoot (RoDrWtSh), canopy green area per meter square at anthesis (GA An) and NDVI at anthesis (NDVI) in 2019. Independent variables selected in the analyses contributed significantly to the models. (DOCX) [file pone.0242472.s006.docx]

**S6 Table.** Stepwise multi-linear regression with grain yield (GY) as the dependent variable showing the best model for selected traits in irrigated (IR) and semiarid (SA) conditions in 2018 and 2019: Grain yield (GY), root surface area (RoSuAr), root diameter (RoDiM), root volume (RoVol), NDVI at anthesis (NDVI), NDVI senescence start (SenSt), NDVI senescence duration (SenDu) in 2018 and root angle (RoAng), root diameter (RoDiM), root dry weight per shoot (RoDrWtSh), canopy green area per meter square at anthesis (GA An) and NDVI at anthesis (NDVI) in 2019. Independent variables selected in the analyses contributed significantly to the models.

| Treatment/Year | Dependent Variable | Independent Variable | R^2^ | No of traits | Best Model |
| --- | --- | --- | --- | --- | --- |
| 18IR | GY | RoSuAr, RoVol, RoDiM, NDVI, SenSt, SenDu | 0.13 | 1 | SenSt |
|  |  |  | 0.29 | 2 | SenSt, NDVI, |
|  |  |  | 0.32 | 3 | SenSt, NDVI, RoDiM |
| 18SA | GY | RoSuAr, RoVol, RoDiM, NDVI, SenSt, SenDu | 0.10 | 1 | NDVI |
|  |  |  | 0.16 | 2 | NDVI, RoSuAr, |
|  |  |  | 0.17 | 3 | NDVI, RoSuAr, SenDu |
| 19IR | GY | RoAng, RoDiM, RoNoSh, GA An, NDVI An | 0.16 | 1 | GA An |
|  |  |  | 0.22 | 2 | GA An, RoAng |
|  |  |  | 0.24 | 3 | GA An, RoAng, RoNoSh |
| 19SA | GY | RoAng, RoDiM, RoNoSh, GA An, NDVI An | 0.33 | 1 | GA An |
|  |  |  | 0.39 | 2 | GA An, RoNoSh |
|  |  |  | 0.41 | 3 | GA An, RoNoSh, RoAng |

**S6 Table.** Percent yield reduction under SA conditions for 50 genotypes (Mean of 2018 and 2019).
