# Supplementary figures and images for: Root and canopy traits and adaptability genes explain drought tolerance responses in winter wheat
Source: PLoS One. 2021 Apr 5;16(4):e0242472. doi: 10.1371/journal.pone.0242472 (PMC8021186; doi:10.1371/journal.pone.0242472)

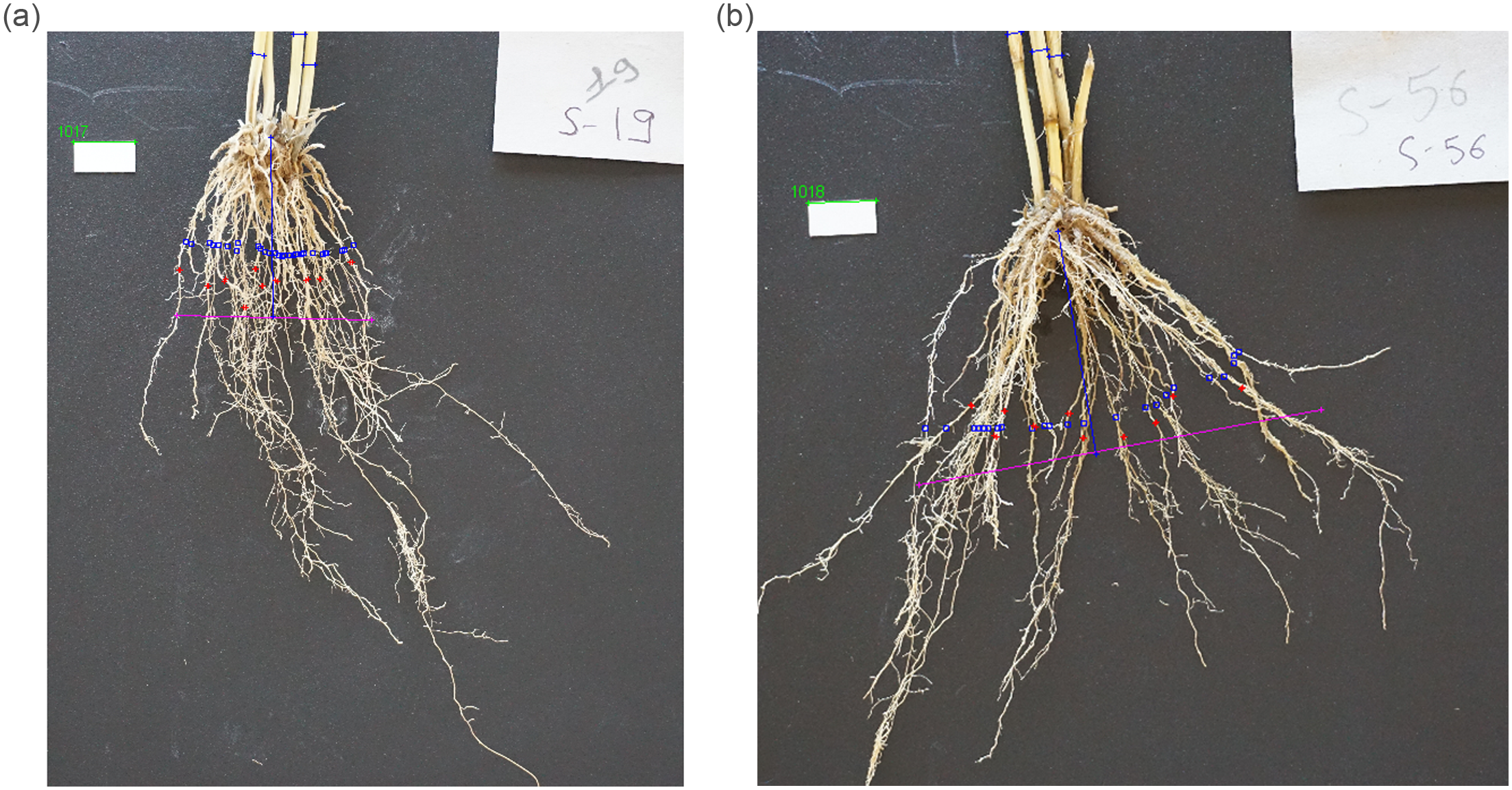

Supplement: S1 Fig — An example image of the entire wheat root crown showing genetic variation in root system, rectangular scale (top left), and plot ID tag (top right). Some of the measured features include stem width (top green line), system width (bottom pink line), number of nodal roots (middle blue points), and distance from root origin to system width lines (vertical blue line). (TIF) [file pone.0242472.s007.tif]
